# Pediatric Traumatic Brain Injury in the Middle East and North Africa Region: A Systematic Review and Meta-Analysis to Assess Characteristics, Mechanisms, and Risk Factors

**DOI:** 10.1089/neur.2023.0007

**Published:** 2023-10-17

**Authors:** Samar Al-Hajj, Sarah H. Farran, Batoul Dekmak, Layal Hneiny, Hussein Abou Abbas, Aya Hassoun, Nadine Youness, Sarah Ghalayini, Nour Abou Khalil, Fiona Lecky, Shima Shahjouieh, Layal Ghamlouche, Zainab Nasrallah, Firas Kobeissy

**Affiliations:** ^1^ Department of Epidemiology and Population Health, Faculty of Health Sciences, American University of Beirut, Beirut, Lebanon; ^2^ Faculty of Medicine, American University of Beirut, Beirut, Lebanon; ^3^ Saab Medical Library, American University of Beirut, Beirut, Lebanon; ^4^ Department of Internal Medicine, American University of Beirut, Beirut, Lebanon; ^5^ School of Public Health, University of Saskatchewan, Saskatoon, Saskatchewan, Canada; ^6^ Centre for Urgent and Emergency Care Research, University of Sheffield, Sheffield, United Kingdom; ^7^ Department of Neurosurgery, University of New Mexico, New Mexico; ^8^ Department of Biochemistry and Molecular Biology Faculty of Medicine, American University of Beirut, Beirut, Lebanon; ^9^ Department of Neurobiology, Center for Neurotrauma, Multiomics & Biomarkers (CNMB), Neuroscience Institute, Morehouse School of Medicine, Atlanta, Georgia, USA

**Keywords:** MENA, meta-analysis, pediatric traumatic brain injury, systemic review

## Abstract

Pediatric traumatic brain injury (pTBI) represents a major cause of child injuries in the Middle East and North Africa (MENA) region. This review aims to assess pTBIs in the MENA region and reports their clinical severity and outcomes. A search was conducted using major electronic databases, including Medline/Ovid, PubMed, EMBASE, Web of Science, and SCOPUS. Abstracts were screened independently and in duplicate to detect original research. The objective and study findings for each article were recorded, along with the mechanism of pTBI, patient age and sex, injury assessment tool(s) used, and outcome. A total of 1345 articles were retrieved, of which 152 met the criteria for full-text review, and 32 were included in this review. Males predominantly suffered from pTBIs (78%). Motor vehicle accidents, followed by child abuse, were the leading causes of pTBI. Overall, 0.39% of cases were mild, 0.58% moderate, 16.25% severe, and 82.27% unclassified. The mortality rate was 13.11%. Most studies used the computed tomography scan, Glasgow Coma Scale, Abbreviated Injury Scale, and Injury Severity Score as investigation methods. This review reports on the alarming rate of child-abuse–related pTBI and offers further understanding of pTBI-associated risk factors and insight into the development of strategies to reduce their occurrence, as well as policies to promote child well-being.

## Introduction

Pediatric traumatic brain injury (pTBI) is a high-priority health issue owing to the serious disability and mortality rates it inflicts on persons worldwide.^1–3^ Traumatic brain injury (TBI) is defined as damage to the brain after a head trauma (e.g., violent hit to the head, penetrating object to skull/brain tissues).^4^ Whereas all age groups suffer adversely from TBIs, children are at an increased risk for TBI-related hospitalization, disability, and death because of their physiological and anatomical vulnerability and propensity to sustain major injuries.^1,2,4^ Children have relatively weaker necks and torsos compared to adults; thus, any minor force may result in a serious injury, especially if inflicted in the head region and affecting the brain at its early developmental stages.^5^ Besides, a child's developing brain is smaller and more pliable with higher water content compared to the adult brain, which might lead to greater vulnerability to injury and different patterns of child injuries. Children have softer skulls than adults, with a thinner layer of protective fluid making them more susceptible to damage from even minor impacts. Myelination, the process by which nerve fibers are coated with myelin to increase the speed of electrical impulses, is incomplete in a child's developing brain, which can affect the severity and long-term effects of pTBI.^6^

The global burden of TBI is growing substantially. Further evidence is shown in low- and middle-income countries (LMICs), which suffer 3 times more TBI incidences compared to high-income countries (HICs).^1,2^ The United States reported >145,000 annual cases of children and adolescents (ages 0–19) suffering from long-term cognitive, physical, and behavioral effects attributable to TBI.^7^ In the Middle East region, the median TBI incidence rate per capita is ∼45 per 100,000 population, which is ∼3 times higher than that in HICs.^8^ According to the Global Burden of Disease Study 2016, there were 27 million new cases of TBI globally in 2016.^9^

TBI severity classification ranges from mild, moderate to severe and incorporates several parameters, including the Glasgow Coma Scale (GCS), level of consciousness, anatomical injuries on computed tomography (CT) scan with the Abbreviated Injury Scale (AIS), International Classification of Diseases, or Marshall classification of structural imaging, and occurrence of post-traumatic amnesia.^3^ Acute TBI symptoms largely depend on TBI severity and include loss of consciousness or other neurological deficits, headache, and vomiting. Patients may be left with long-term physical, cognitive, or emotional impairments.^4^ The reported etiology of pTBI varies from road traffic injuries to sports injuries to violence and child abuse. The Eastern Mediterranean Region sustains—in addition to routine TBIs caused by falls, car crashes, sports, and work-related injuries—a series of violence-related injuries because of ongoing violence, wars, and regional conflicts. The region's long history of violence and wars has also taken its toll on the pediatric population. A retrospective review of the Joint Theater Trauma Registry revealed that between 2004 and 2012, a total of 647 children were treated for severe isolated TBIs at a combat support hospital in Iraq and Afghanistan, of which 51% underwent a craniotomy or a craniectomy.^10^

Previous regional studies have examined the epidemiology of TBI in the Middle East and North African (MENA) region.^11,12^ These existing studies were limited in their scope and approach, focusing on a broad population, district, or TBI type.^11^ Incidence and distribution of pTBI by age, sex, and region in the Middle East remain understudied, with several gaps in basic, epidemiological, clinical, and translational TBI-related research. Research in the field of pTBI is crucial to provide awareness and inform policies for age-appropriate interventions to decrease pTBI rates and improve its long-term outcomes. This study aims to conduct a systematic review of the literature and a meta-analysis to assess the epidemiological patterns, common mechanisms, types, and clinical outcomes of pTBI in the MENA region. This meta-analysis will be the first to assess, across all study designs, the characteristics, mechanisms, severity, and clinical outcomes among the TBI pediatric population in the MENA region.

## Methods

We prepared and reported the present study according to the Preferred Reporting Items for Systematic Reviews and Meta-Analyses (PRISMA), Meta-analysis of Observational Studies in Epidemiology (MOOSE), and Enhancing the QUAlity and Transparency Of health Research (EQUATOR) guidelines.

### Search strategy and information sources

A systematic research was conducted using the following databases: Medline by way of Ovid, PubMed, EMBASE, Web of Science (WOS), SCOPUS, Current Index to Nursing & Allied Health Literature (CINHAL), Cochrane, and Global Health Library (search protocol available in Supplementary Appendix SA). Studies were retrieved from the inception of databases until March 2022, with no restriction on language or publication type. Key index and MeSH/EMTREE terms to search for TBI included: “Brain trauma”; “Brain injuries;” and “Brain concussion.” Key terms for literature published from the MENA region included “Middle East,” “Northern Africa,” and a list of all Arabic-speaking countries. Our search was narrowed to the pediatric age group (<18 years), which we defined by terms such as “child,” “infant,” “adolescent,” “pediatric,” and several more. The complete search strategy used to generate the results is presented in the Supplementary Material.

### Inclusion/exclusion criteria

Retrieved studies were eligible if they 1) investigated patients (outpatient or inpatient) with an admission diagnosis of pTBI confirmed by an attending physician and 2) were unpublished or published cross-sectional, case-control, prospective, retrospective, and clinical trial studies. Articles were excluded if they were abstract-only reports, case series, or conference lectures and if they recruited patients from outside the MENA region.

Studies investigating non-Arabic-speaking countries—such as Turkey, Iran, and Israel—were excluded. The decision to exclude non-Arabic-speaking countries was based on cultural and social differences among these Arabic and non-Arabic-speaking countries. The included Arabic-speaking countries are all governed by the regulations of the Council of the League of Arab States, which comprises 22 Arabic-speaking countries that share similar educational and medical programs. Therefore, it was assumed that the response to national health issues such as TBI would be homogenous among these countries. Moreover, the decision to use the same exclusion/inclusion criteria as our previous study published in the *Journal of Neuroepidemiology* was made to ensure the consistency and comparability of the results between the adult and pediatric TBI studies.^11^ Additionally, several studies have been conducted in the MENA region with a specific focus on Arab countries, supporting the rationale for the exclusion of non-Arabic-speaking countries.

Further, we excluded studies that did not characterize the mechanism of injury, such as motor vehicle crashes, falls, military, assault/violence, and sports. Two co-authors (S.A. and L.H.) independently screened the recruited titles and abstracts based on a pre-defined protocol. Discrepancies were resolved by a discussion with a third investigator (F.K.).

### Data abstraction and analysis

Two co-authors (S.F. and B.D.) independently abstracted the study variables and key findings onto a customized data extraction sheet in Excel. Details abstracted from each article include title, author, publication year, country, source population, sample size, study objective, key findings, age groups, sex, outcome measures reported (incidence, prevalence, severity, mortality, case fatality, morbidity, disability, recovery status, etc.), anatomical location of the injury, mechanism of the injury, the abuser (if child abuse), injury classification (open, penetrating, blunt, etc.), severity (documented severity, GCS, Functional Independence Measurement [FIM], Disability Rating Scale [DRS], cerebral CT scan, Injury Severity Score [ISS], AIS score, and Revised Trauma Score), case ascertainment method, surgical intervention performed, complications (intracranial hemorrhage, infections, death, etc.), long-term outcomes, length of hospital stay, intensive care unit (ICU) admissions, rehabilitation, and costs.

### Synthesis of the evidence

Because of the scarcity of available TBI studies in the MENA region, all existing TBI studies were included in this review. We carried out a descriptive summary of the findings (Table 1). Where appropriate, we calculated the mean or proportion of the variable of interest using the combined sample. We analyzed the quality of the selected articles using STROBE (Strengthening the Reporting of Observational Studies in Epidemiology), which assesses 22 key items that should be present in the titles, abstracts, introductions, methodologies, results, and discussions of a cross-sectional survey or case-control and cohort observational studies. From this analysis, we obtained the STROBE results for each study, displayed in Table 2, where the articles that included 0–7 items were considered low quality, 8–14 items were intermediate quality, and 15–22 items were high quality.

**Table 1.** Summary of Aims and Key Findings of Included Studies

Name of the author(s)	Year of publication	Country of study	Study aims	Study key findings	Sample size	Mean age (years) of subjects
Hawamdeh et al.^31^	2011	Palestine	Determine the disability aspects and its levels among patients with TBI and their caregivers in Gaza Strip	The worst performance of patients with TBI according to the FIM was recorded in bowel and bladder control (40.1% and 35.8%, respectively). The DRS questionnaire revealed that employability established the area of greatest disability for patients with TBI (69.33%).	100	
Bahloul et al.^21^	2009	Tunisia	Determine epidemiological, causes, clinical, and para clinical manifestations and outcomes in children with a TBI in south Tunisia	The predominance of male patients with a sex ratio of 2.21. The most observed lesions were meningeal hemorrhage (35.2%) and cerebral contusion (34.5%). Secondary systemic insults were observed in 377 children (83%) who are essentially involved in traffic crashes.	454	7.2 ± 3.8 (<15 years)
Jamous et al.^34^	2009	Jordan	Report the experience in non-operative management of acute EDH in children with a mild TBI	Proved, in the population of the study, the safety and success of conservative management in patients with normal or minimal symptoms and significant EDH on brain CT scans. Pediatric EDH can be managed non-operatively. The pronounced increase in the number of CT examinations for patients with TBIs has resulted in a greater proportion of EDH detected in conscious patients.	6	11.4
Alhabdan et al.^18^	2013	KSA	Determine the characteristics, etiology, and outcome of TBI in the pediatric population and compare findings to international figures	Increased association of obesity with worse injury severity and prolonged length of hospital stay. Obesity was associated with worse TBIs, which could be attributed to the high incidence of MVCs in the current study.	1219	8.6
El-Menyar et al.^12^	2017	Qatar	Describe the hospital-based epidemiological characteristics, injury mechanisms, clinical presentation, and outcomes of pTBI and analyze key characteristics and determinants of pTBI	1 in 6 victims of TBI in Qatar is a child. The leading mechanism of injury and the outcomes of pTBI are age dependent. The most affected group is teenagers (40%), followed by infants/toddlers (23%). Two thirds of the cohort has severe pTBI. Fifth, males predominate among the victims, but the sex difference is narrowing.	167	10.6
Bahloul et al.^20^	2009	Tunisia	Determine predictive factors of mortality among children after TBI	In Tunisia, TBI is a frequent cause of hospital admission and is most often attributable to RTCs. Short-term prognosis is poor, with a high mortality rate (24.3%), and is influenced by demographic, clinical, radiological, and biological factors. Univariate analysis showed that low PTS on admission, high ISS or PRISM, presence of shock or meningeal hemorrhage or bilateral mydriasis, and serum glucose >10 mmol/L were associated with mortality rate. Multi-variate analysis showed that factors associated with a poor prognosis were PRISM >20 and bilateral mydriasis on admission.	222	7.5 (<15 years)
Mehmood et al.^40^	2018	Oman	Describes the epidemiology and risk factors for childhood injuries (0–15 years of age), in two hospitals in Oman	Patients suffering from head injuries (RR, 8.8; 95% CI, 4.9–15.3) or being involved in a burn injury (RR, 1.5; 95% CI, 0.3–7.5) were at increased risk of undergoing surgical treatment.	344	0–15 years
Grivna et al.^28^	2013	UAE	Assess causes and determinants for traffic-related injuries during childhood and youth (<19 years) and its value for prevention	Traffic injuries represented 40% (*n* = 193) of injuries to 0- to 19-year-olds, followed by falls (39%). The most frequent anatomical location of traffic-related injuries was the head, accounting for nearly 40% of injuries. The proportion of males with severe TBIs of AIS ≥3 was >4 times greater than the proportion of females (27% vs. 6%).	116	
Crankson et al.^24^	2006	KSA	Analyze children with motor vehicle injuries to discuss nation-wide preventive programs required to target the use of seatbelts and helmets and dangerous driving practices	Motor vehicle injuries accounted for ∼42% of all pediatric traumas. The most common injuries were to the head and extremities. TBI is the most common cause of morbidity and mortality. The risk factors associated with a high volume of motor vehicle injuries in children include males, 1–8 years, and pedestrians. This study suggests that nation-wide programs should target the use of seatbelts and helmets and dangerous driving practices.	378	5.2 (<12 years)
Hefny et al.^32^	2015	UAE	Assess the anatomical distribution, severity, and outcome of hospitalized trauma pedestrian patients in Al-Ain, UAE	Mortality of pedestrian injured patients in the UAE is high. The lower limb was the most injured region, followed by the head. Severe TBI was the main cause of death.	71	
Grivna et al.^29^	2013	UAE	Assess the mechanism of RTCs, use of safety devices, and outcome of hospitalized pediatric and youth RTC injured patients	The most frequent anatomical location of injury was the head (42%), followed by the extremities. Male drivers and UAE nationals were at high risk of RTC as drivers and as motorcyclists. The ejection rate was high because safety restraint use was extremely low in our community.	132	0–19 years

KSA, Kingdom of Saudi Arabia; UAE, United Arab Emirates; TBI, traumatic brain injury; EDH, epidural hematoma; pTBI, pediatric traumatic brain injury; RTCs, road traffic collisions; FIM, Functional Independence Measurement; DRS, Disability Rating Scale; CT, computed tomography; MVCs, motor vehicle crashes; PTS, Pediatric Trauma Score; ISS, Injury Severity Score; PRISM, Paediatric Risk of Mortality; RR, risk ratio; CI, confidence interval.

**Table 2.** STROBE Analysis of the Studies

Author (year)	Country, duration	Study sample size and inclusion/exclusion criteria	Injury severity	Incidence/mortality	STROBE criteria
Hawamdeh et al. (2011)^31^	Palestine, 2000–2007	Sample size: 100 Inclusion criteria: TBI patients 5–69 years of age who were admitted to the El-Wafa medical rehabilitation hospital from 2000 to 2007, severity of TBI graded as moderate (9–12) according to the GCS, TBI patients were home residents for at least 6 months before inclusion in the study, male and female caregivers 18–50 years of age, caregivers free from severe medical problems or disability	N/A	N/A	The study design was only mentioned in the abstract and was not elaborated on in the article. The article only mentioned that the study took place from 2000 to 2007 at a particular hospital in the Gaza Strip. The study mentioned that convenience sampling was used without further elaboration and stated the eligibility criteria. The data sources/measurements were well presented. An explanation of how the study size was arrived at was presented.
Bahloul et al. (2009)^21^	Tunisia, 1997–2004	Sample size: 454 Inclusion criteria: children with TBI admitted to the ICU of a university hospital (Sfax-Tunisia) during 1997–2004	GCS = 8 ± 3 points	82 of 454 cases	Complete STROBE criteria
Jamous et al. (2009)^34^	Jordan, August 2003 and October 2007	Sample size: 6 children Inclusion criteria: patients with conservatively treated EDH at the Department of Neurosurgery, King Abdulla University Hospital, Irbid, Jordan, between August 2003 and October 2007	GCS = 15 in 4 patients and GCS = 14 in the other 2 patients	N/A	For study design, the study only mentioned that the charts were reviewed retrospectively.
Alhabdan et al. (2013)^18^	KSA, 2001–2009	Sample size: 1219 Inclusion criteria: all consecutive patients at ≤18 years who were identified through the King Abdulaziz Medical City Trauma Registry (KAMC-TR) with a diagnosis of TBI	Mean ISS was 16.6 (range 1–75), and median GCS was 11 (range 3–15).	After assessment and resuscitation in the ED, 7.5% died, 33.6% were admitted to the critical care unit, and 6.3% underwent surgery. The overall mortality rate was 14.7%; half died on arrival.	Complete STOBE criteria The study design was a retrospective cohort study that was mentioned in the article and methods. The period of exposure was 2001–2009. The study setting was also included. The study mentioned the eligibility criteria as well as the sources and methods of selection of participants and methods of follow-up. The study also explained how the study size was arrived at.
El-Menyar et al. (2017)^8^	Qatar, 2010–2014	Sample size: 167 Inclusion criteria: pTBI cases ≤18 years of age whose names were present in the trauma registry of the Hamad Trauma Center (HTC) from January 1, 2010 to December 31, 2014	Based on the GCS at ED, severity of the injury was mild (12.2%), moderate (22.6%), and severe pTBI (65.2%).	Overall mortality was 13.3%.	Complete STROBE criteria
Mehmood et al. (2018)^40^	Oman, November 2014 to April 2015	Sample size: 795 Inclusion criteria: All patients between 0 and 15 years with a diagnosis of injury/trauma admitted to the hospital, including those referred or transferred in, and those who had trauma team activation in, the ED were included in the analysis.	Not available	Not available	Complete STROBE criteria
Grivna et al. (2013)^28^	UAE, 2003–2006	Sample size: 193 (total), 117 (TBI) Inclusion criteria: children and youth with traffic injuries who were admitted for more than 24 h at surgical wards of the main trauma hospital in the Al-Ain region during36 months (2003–2006)	Median severity of all injuries was 4, 16% of injuries were severe with ISS ≥15; head AIS: mean = 1.7, median = 1	Not available	The study design should be elaborated on.
Crankson (2006)^24^	KSA, January 1994 to December 2003	Sample size: 664 (total), 378 with TBI Inclusion criteria: all children who are ≤12 years old and were admitted and treated at King Fahad National Guard Hospital, Riyadh from January 1994 to December 2003 with the clinical diagnosis of motor vehicle injury, including the external cause of injury codes (E-codes)	Of 378 children, 50% had a mild pTBI, 26% had moderate pTBI, and 24% had severe pTBI.	Mortality attributable to TBI: 30 cases	Complete STROBE criteria
Hefny et al. (2015)^32^	UAE, March 2003 to October 2007	Sample size: 318 (total population) 71 (children, <18 years old) Inclusion criteria: all trauma pedestrian patients who were involved with RTC and were admitted to Al-Ain Hospital for more than 24 h or who died in the hospital after arrival during March 2003 to October 2007	Median GCS = 15 for all patients who survived and for all injuries Median GCS = 3 for all patients who died and for all injuries Median ISS = 5 for all patients who survived and for all injuries Median ISS = 22 for all patients who died and for all injuries	30 of all populations with all types of injuries	Complete STROBE criteria
Grivna et al. (2013)^29^	UAE, April 2006 to October 2007	Sample size: 254 Inclusion criteria: All injured children and youth 0- to 19-year-olds who were admitted to Al-Ain City's two major trauma centers or who died after arrival to these hospitals after being involved in an RTC from April 2006 to October 2007 were prospectively studied.	GCS = 15, ISS = 5, and AIS depends on injury (not for TBI).	Mortality among vehicle occupants was 4.1% (not for TBI).	Complete STROBE criteria
Alnasser et al. (2012)^44^	KSA, 2001–2009	Sample size: 3766 Inclusion criteria: all pediatric patients from 0 to 18 years of age, who were involved in trauma between May 2001 and 2009 and registered in the King Abdulaziz Medical City Trauma Registry		2.8% death on arrival and 4% death in the hospital	Complete STROBE criteria
Kariyattil and Muthukuttiparambil (2012)^46^	Oman, 2012	Sample size: 1 Inclusion criteria: case study	GCS on arrival = 11	0	Case study: STROBE is not applicable.
Al-Adawi et al. (2012)^14^	Oman: The exact study period was not mentioned, but it was mentioned that the interviews were conducted over 1 year.	Sample size: 2 (adults) Inclusion criteria: case study		0	Case study: The study thoroughly explained the study design, setting, and participants. The exact study dates were not mentioned.
McGuigan et al. (2007)^39^	Iraq, January 1 to December 31, 2004	Sample size: 99 Inclusion criteria: patients who are ≤17 years who were treated in the combat support hospital from January 1 to December 31, 2004	ISS = 11.6 for all injuries Head and neck AIS = 0.99	Total mortality is 9% (23% TBI mortality)	Complete STROBE criteria
Celikel et al. (2015)^23^	Syria, January 2012 and August 2014	Sample size: 140 dead children, among which 42 had pTBI Inclusion criteria: post-mortem examination and autopsy reports of forensic deaths of children <18 years from Hatay, a Syrian neighborhood city of Turkey, between January 2012 and August 2014	All cases are dead. ISS for head and neck is 37.5.	All cases are dead.	Complete STROBE criteria
Klimo et al. (2015)^10^	Iraq/Afghanistan, 2004–2012	Sample size: 268 during the Operations Iraqi Freedom (OIF) Inclusion criteria: children (<18 years old) in the Joint Theater Trauma Registry with isolated TBI (defined as an AIS Severity Code >3) and treated at a U.S. combat support hospital in Iraq or Afghanistan from January 1, 2004 through December 31, 2012	All are severe, defined as AIS >3; GSC on admission = 7	pTBI mortality: 25.7% (69 cases) in Iraq	Complete STROBE criteria
Creamer et al. (2009)^25^	Iraq/Afghanistan	Sample size: 221 in Iraq and 262 in Afghanistan Inclusion criteria: all available data on pediatric (children from birth through the age of 17) admissions entered into the PASBA database through the end of October 2007	Not available	Total deaths in Iraq: 60 TBI deaths in Iraq: 25 (41% of all deaths)	The study design, participants, data sources and measurement, and study size should be elaborated upon. The study design was mentioned briefly. The inclusion criteria were not clearly stated. The exact dates of data collection were not mentioned or clearly stated.
Er et al. (2017)^27^	Syria, July 2013 to October 2014	Sample size: 185 children (pTBI: 156) Inclusion criteria: patients who were classified in the hospital data-registration system using the SCW-specific code and who did not meet any of the exclusion criteria, which were being admitted to a hospital department other than the ED, having no electronic data available, and having file records from which data were missing	Median ISS was 16 for all patients.	Total mortality among children: 7 cases: 4 from isolated brain injury, 1 from the brain and abdominal injury, and 2 from burns	Complete STROBE criteria
Edwards et al. (2012)^26^	Iraq/Afghanistan, 2002–2010	Sample size: 1822: 1205 below 15 years AND 617 between 15 and 20 Inclusion criteria: civilian patients admitted to military treatment facilities (Role 3) in Iraq and Afghanistan from 2002 to 2010 with injuries because of an explosive device	ISS: 1) For all type of injuries: mean (SD) ISS <7 years, 13.5 (10.7); 8–14 years, 14.9 (12.6); 15–20 years, 12.8 (11.0); above 20 years, 13.3 (11.5). 2) ISS >15 in 690 children; 37.8%. AIS: 1) Severe head and neck injury (AIS >3): 395 children; 21.7% of the population. 2) For adults and children casualties (%) BR1: Head/cervical spine scale 0: 3511 (71.7) scale 1–2: 386 (7.9) scale 3–6: 1000 (20.4)	1) Mortality in children from all injuries: 119 children; 6.5%. 2) The overall mortality rate for children <15 is 7.8%. 3) Head/cervical spine injury mortality odds ratio is 1.15.	The study design was not mentioned throughout the article. Also, further elaboration on the participants is needed.
Martin et al. (2010)^38^	Iraq, 2007	Sample size: 42 Inclusion criteria: individuals 17 years old or younger			
Kemp et al. (2008)^45^	Until May 2007	No. of studies: 32 Inclusion criteria: comparative studies of fracture at different bony sites, sustained in physical abuse and from other causes, in children <18 years old	N/A	N/A	Systematic review STROBE N/A
Al-Ateeqi et al. (2002)^16^	Kuwait, 1991–1998	Sample size: 16 Inclusion criteria: children who showed evidence of abuse among the 60,640 medical records of children admitted to Al-Amiri and Mubarak Al-Kabeer hospitals, Kuwait, between 1991 and 1998	N/A	Total mortality is 12% (2 of 16); pTBI mortality is 20% (1 of 5 cases).	The eligibility criteria are not clearly stated; however, it is evident in the context of the study. The data sources/measurements are not clearly stated.
Jawadi et al. (2019)^35^	KSA, 2009–2015	Sample size: 56 Inclusion criteria: all confirmed cases for children (≤14 years of age) of non-accidental fractures registered in the National Family Safety Program Registry at King Abdulaziz Medical City, Riyadh, between 2009 and 2015 with available radiographs	N/A	Three (5.4%) children died because of abuse.	Complete STROBE criteria
Al-Mahroos et al. (2012)^17^	Bahrein, January 2000 to December 2009	Sample size: 237 Inclusion criteria: The study included all children from birth to <18 years of age who were evaluated by the Child Protection Unit for physical abuse, with or without sexual abuse from January 2000 to December 2009. Cases with sexual abuse only were excluded because the focus of this report was child physical abuse. Cases that had signs and symptoms indicative of diseases mimicking child abuse or incidental injuries were excluded.	N/A	TBIs were observed in 9.7% of the cases (23 cases).	Complete STROBE criteria
Al-Mahroos et al. (2011)^30^	Bahrein, 2000–2009	Sample size: 36 Inclusion criteria: Only children who sustained skeletal fracture as a result of child abuse were included in the study	Severe	17% (4 of 23 cases)	Complete STROBE criteria
Alsaif et al. (2013)^19^	Egypt, 2006–2010	Sample size: 60 Inclusion criteria: children cases of child homicides of the death records in Zeinhom morgue between 2006 and 2010	Dead cases	The mortality rate was 100%; 73% of internal injuries were mostly intracranial (41 cases).	Data sources/measurements can be elaborated on. Injury severity was not mentioned.
Nazer et al. (1988)^42^	Jordan, 1988	Sample size: 2 Inclusion criteria: a case study	N/A	0%	Case study: STROBE is not applicable.
Bener et al. (2005)^22^	Qatar, January 1992 to December 2003	Sample size: 275 Inclusion criteria: all incidents, injuries, and deaths among children camel jockeys 5–15 years of age for the period between January 1992 and December 2003	AIS minor/maximum depending on the case	TBIs were observed in 20.7% of the cases (57 cases); 3 cases died of all injuries.	Complete STROBE criteria
Nawaz et al. (2005)^41^	UAE, January 1992 to December 2002	Sample size: 78 Inclusion criteria: all children admitted to our hospital with the diagnosis of camel-related injuries	For TBI, 27 cases had mild injuries and 17 had moderate-to-severe TBIs.	Mortality rate: 0% TBI incidence: 56.4% (44 cases)	Complete STROBE criteria
Hoz et al. (2019)^33^	Iraq, January 2015 to January 2017	Sample size: 29 Inclusion criteria: patients 9 years old or younger who sustained head injuries due to metallic ceiling fans admitted to the Emergency Department of Neurosurgery Teaching Hospital in Baghdad, Iraq, between January 2015 and January 2017	GCS = 3–15	3% (1 case)	Complete STROBE criteria
Parchani et al. (2013)^43^	Qatar, 2008–2011	Sample size: 107 Inclusion criteria: all patients with a TBI who sustained recreation-related injuries and were presented to the only level I trauma center in Qatar and admitted to the trauma ICU at the Hamad General Hospital between January 2008 and July 2011	Median ISS = 10 Median GCS = 15 Median head AIS = 3	The overall mortality rate is 7%.	Complete STROBE criteria

STROBE, Strengthening the Reporting of Observational Studies in Epidemiology; KSA, Kingdom of Saudi Arabia; UAE, United Arab Emirates; TBI, traumatic brain injury; GCS, Glasgow Coma Scale; ICU, intensive care unit; EDH, epidural hematoma; RTC, road traffic collision; pTBI, pediatric TBI; PASBA, Patient Administration System and Biostatistics Activity database; SCW, Syrian Civil War; ED, emergency department; AIS, Abbreviated Injury Scale; N/A, not applicable; ISS, Injury Severity Score; SD, standard deviation.

### Study appraisal

#### Meta-analysis

In this meta-analysis, summary estimates for mortality rates were generated using the meta-analysis approach. Sources of heterogeneity were explored using subgroup analyses. Subgroup analyses were considered according to the cause of injuries and country's income level. No transformations for prevalence rates were done. We used the random-effects model to report pooled prevalence rates. A random intercept model was used to account for heterogeneity.

### Statistical analysis

To report pooled prevalence rates, a random-effects model was used in this meta-analysis. The random-effects variable in this meta-analysis is the variation in prevalence rates across studies. The fixed-effects included in the model were cause of injuries and the country's income level, which were used to explore sources of heterogeneity and conduct subgroup analyses. No interactions were considered, and a random intercept model was used.

## Results

**FIG. 1.**
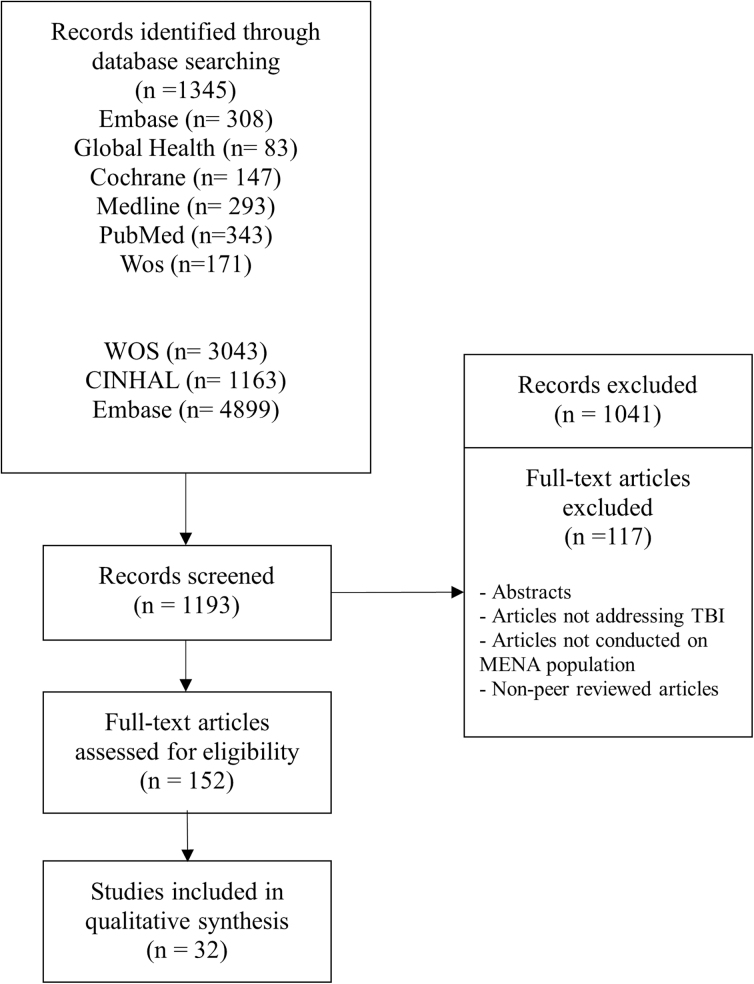
PRISMA flow diagram for pediatric TBI in the MENA region showing the selection process, number of documents screened, evaluated for eligibility, and included and reasons for exclusion after screening. CINAHL, Current Index to Nursing & Allied Health Literature; MENA, Middle East and North Africa; PRISMA, Preferred Reporting Items for Systematic Reviews and Meta-Analyses; TBI, traumatic brain injury; WOS, Web of Science.

**FIG. 2.**
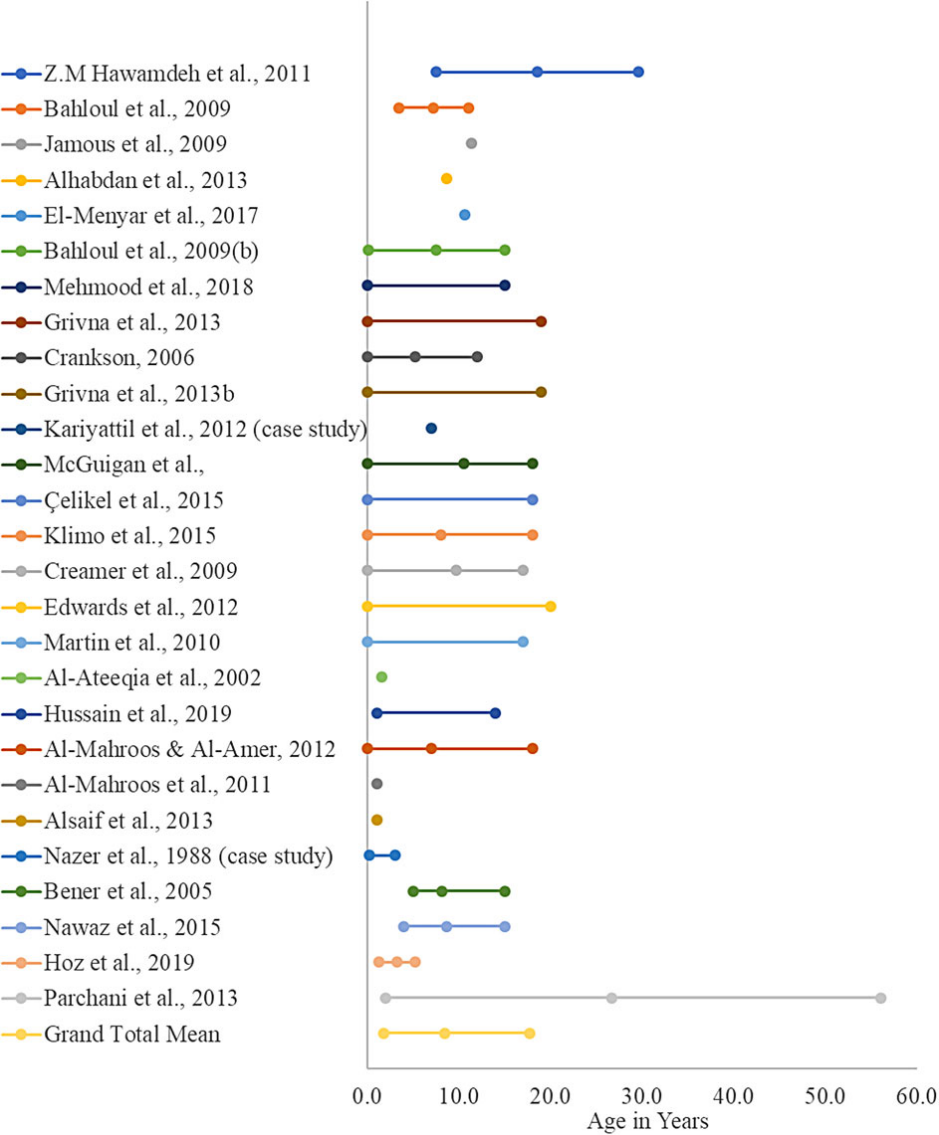
Age distribution of traumatic brain injuries (years) across different studies conducted in different countries within the MENA region. MENA, Middle East and North Africa.

**FIG. 3.**
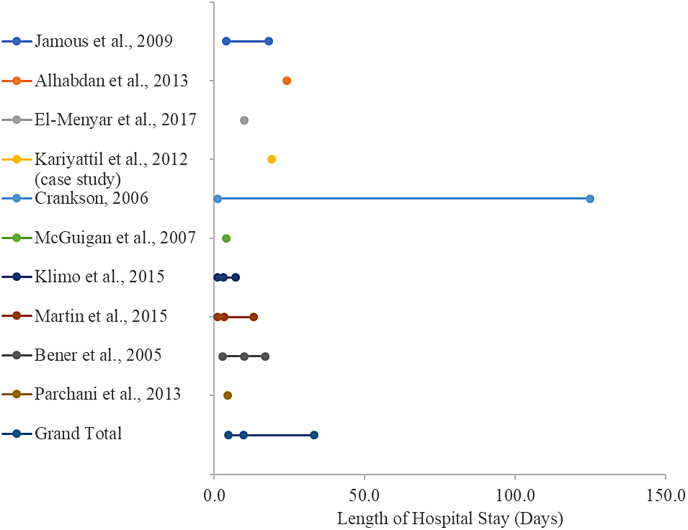
Length of hospital stay distribution of pediatric traumatic brain injuries (day).

The search strategy resulted in 1345 references, of which 152 met the criteria for full-text review. Based on the eligibility criteria, 117 were excluded and the remaining 32 articles were used to review and synthesize findings (refer to PRISMA; Fig. 1).^8,10-44^ Age distribution and hospital length of stay (day) of pediatric traumatic brain injury are shown in Figures 2 and 3.

### Regional distribution

As reported in Table 3, studies were distributed across the MENA region, with most findings originating from Iraq for a total of six articles (two of which also included Afghanistan), followed by the United Arab Emirates (UAE) and Kingdom of Saudi Arabia (KSA), with four each. Qatar, Bahrain, and Oman presented three studies each, whereas two were respectively found for Syria, Jordan, and Tunisia. The remaining MENA countries reported one or no studies (Tables 2 and 3).

**Table 3.** Distribution of Studies per Country

Country	No. of articles	Country (HIC/LMIC)
Egypt	1	
Kuwait	1	
Palestine	1	
Jordan	2	
Syria	2	
Tunisia	2	
Bahrain	3	
Oman	3	
KSA	4	HIC
Qatar	3	HIC
UAE	4	HIC
Iraq	6	

KSA, Kingdom of Saudi Arabia; UAE, United Arab Emirates; HIC, high-income country; LMIC, low- to middle-income country.

### Sex distribution

**FIG. 4.**
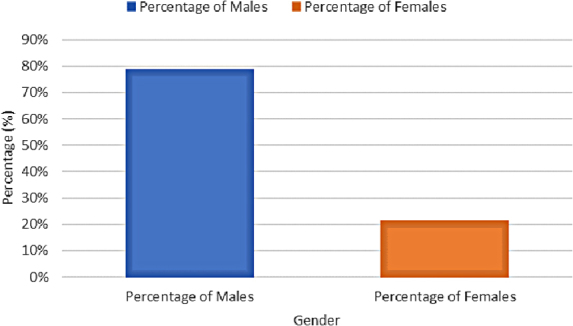
Total sex distribution of pediatric traumatic brain injury cases across included studies within the MENA region (%). MENA, Middle East and North Africa.

Among the 21 articles that reported sex distribution in the pediatric population, most had a higher proportion of male TBI cases. The mean proportion of males was 71.3%, with the median proportion of males being 71.7%. Among the total sample size for studies included in the review, males predominantly suffered from TBIs and accounted for 75.0% (*n* = 2458) of cases (Fig. 4). In Lebanon, the age distribution of TBI victims revealed two peaks—young adults between 18 and 40 years and older adults ≥60 years of age—where males constituted the majority of cases.^13^

### Traumatic brain injury mechanisms or external causes

TBIs resulted from several mechanisms. Seven studies indicated multiple causes of TBI; falls were the leading cause in three studies, motor vehicle accidents (MVAs) in another three, and undefined in the last one.^2,8,11,14,18,37,40^ Seven other studies reported on MVAs only,^20,21,24,28,29,32,44^ and an additional seven studies reported on violence and abuse only.^15–17,19,30,35,42^ Seven studies were military related.^10,13,23,25–27,39^ Three studies looked at recreational and sports activities (including camel racing),^22,41,43^ and one study investigated metallic ceiling-fan injuries.^33^

### Motor vehicle accidents

A total of 15 studies reported MVA-related TBI among children with clinical presentations ranging from mild to severe.^2,8,11,14,18,20,21,24,28,29,32,37,40,44^ Sex-based differences in incidence were observed; in one study, males represented 86% of cases.^4^ Many cases required surgical interventions, such as urgent surgical repair for acute brain herniation and craniotomies. Cerebrospinal fluid otorrhoea and endaural herniation were also repaired using a transcranial, transmastoid, or combined surgical approach. The percentage of TBI mortality attributable to MVAs ranged between 8% and 24%.^3,8^ One article reported 132 pTBI cases and found that only 2% used seatbelts in the vehicle whereas 13% and 0% wore helmets when riding motorcycles and bicycles, respectively.^29^ Assessment tools included CT scan, GCS, ISS, AIS, and the Pediatric Trauma Score (PTS).^2–5,39^ In Oman, TBIs attributable to falls or MVAs were the most commonly recorded injuries among hospitalized children.^43^ MVAs were further indicated as the leading cause of TBIs in Qatar, accounting for 77.3% of cases among 15- to 18-year-old adolescents.^39^ In the same study, underutilization of seatbelts among young vehicle occupants was associated with higher morbidity and mortality, and seatbelt compliance was estimated to reduce severe injuries by 2-fold and mortalities by 4-fold.^39^

### Child abuse and homicide

Child abuse was the second-leading injury mechanism resulting in pTBI. Eight studies included abuse-related pTBI.^15–17,19,27,30,35,42^ Assaults represented 0.4–1.4% of pTBI cases.^3,5^ Among most victims, the abuser was known to the child in 89% of cases and was a parent in 64% of cases.^2,8^ Fall-related pTBI was reported in two articles as a mechanism of child abuse and accounted for nearly 55% of pTBI causes in neglected children.^28,29^ Abusive TBI was also reported in two studies, commonly in children <9 months.^30^ In Saudi Arabia, abusive TBI was the most reported cause of pTBI, accounting for 61% of cases.^35^ Approximately two thirds of pTBI hospital admissions were attributable to caregiver abuse, of which 40% were under intensive care.^28^ Low socioeconomic status (SES) was reported in 53% of cases in one article.^27^ In two studies, most abused victims were boys (60% and 70%),^22,30^ whereas another study found that most homicide victims were mainly girls (53% across all ages and 89% for adolescents 12–18 years).^22^ Two studies reported TBIs as severe.^27,34^ Mortality was an outcome in three studies, ranging from 5.4% to as high as 20%.^28,30,34^ Loss to follow-up was an issue mentioned in two articles.^29,30^

TBI clinical presentations were, namely, contusion and skeletal fractures indicative of structural damage to be considered when diagnosing TBI, in addition to brain injuries and intracranial bleed.^45^ Tools used for TBI screening included x-rays, skeletal films, CT scans, and magnetic resonance images. Some of the complications associated with TBI were subdural hemorrhage, cerebral hemorrhage, retinal hemorrhage, evidence of bruises, skull fractures, localized scalp swelling, subgaleal hematoma, and coma. Surgical interventions were needed in many cases, as mentioned in two studies.^27,34^

### Military

TBI from war and military conflicts was reported in seven studies.^10,13,23,25–27,39^ TBI clinical presentations were mainly penetrating in nature, and the majority were severe in type. There were 69% and 75% more male pediatric cases than females.^26,42^ Death was an outcome in four studies, with mortality rates of 11.3%, 22%, 23%, and 25.7%.^3,12,26,42^ Cause of death was mainly intracranial bleeding and cerebral parenchymal injury.^10^ Tools used for severity assessment were CT, AIS, ISS, and GCS.^3,10,14,26,27,44^ Many cases underwent surgical interventions, such as skeletal fixation, oral/facial operation, soft tissue repair, ocular operation, craniotomy, and decompressive craniectomy, and many children had an intracranial pressure monitor placed.

### Recreational injuries

Two studies reported camel-related TBI,^22,41^ and one reported recreational TBI,^43^ with clinical presentation ranging from mild to severe TBI. Overall, 11% of patients with recreational-related injury and 1 patient (1%) with camel-related injury required a craniotomy. The mortality rate was 3% attributable to camel-related injury and 5% after recreational injury. Approximately 90% of recreational-related TBIs were associated with all-terrain vehicles. Tools used for TBI assessment were CT and AIS, in addition to the GCS.^20,21,25^

### Falls

Fall-related TBIs were reportedly a mechanism in eight articles.^2,8,11,13,14,18,37,40^ In five articles, fall was the primary mechanism and a secondary cause in the remaining three articles.^2,3,5,39,43^ There were no studies exclusively based on falls, and the clinical presentations were unstated. TBI assessment tools included radiography CT scan, x-rays, ISS, AIS, GCS, and PTS.^2,3,5,28,29,39^

### Traumatic brain injury characteristics

Twelve studies reported on the specific characteristics of the brain injury sustained.^15,20–22,24,26,30,34,35,37,43,44^ The most commonly reported injuries were subdural hematomas and intracranial hemorrhage in eight studies each.^15,20,21,30,34,35,41,43^ Of all diagnosed injuries, contusions had the highest mean reported proportion, at 41.3% overall, and a median of 48.3. Table 4 shows the detailed distribution of diagnosed TBI characteristics across all 12 studies.

**Table 4.** Distribution of Studies by Diagnosed Injury Characteristics

Diagnosed injury characteristics	No. of studies reporting the diagnosis
Contusion	5
Subdural hematoma	8
Epidural hematoma	4
Meningeal hematoma	2
Subarachnoid hemorrhage	4
Intraparenchymal hemorrhage	8
Edema	5
Pneumocephalus	5
Diffuse axonal injury	3
Extradural hemorrhage	3
Intraventricular hemorrhage	1
Extra-axial hemorrhage	1
Brain herniation	1

### Traumatic brain injury outcomes

TBI outcomes were evaluated in terms of mortality, severity, and long-term outcomes.

#### Mortality

Mortality refers to the number of TBI-related deaths within a population, and the case-fatality proportion quantifies the number of TBI patients who died because of a sustained brain injury.

**FIG. 5.**
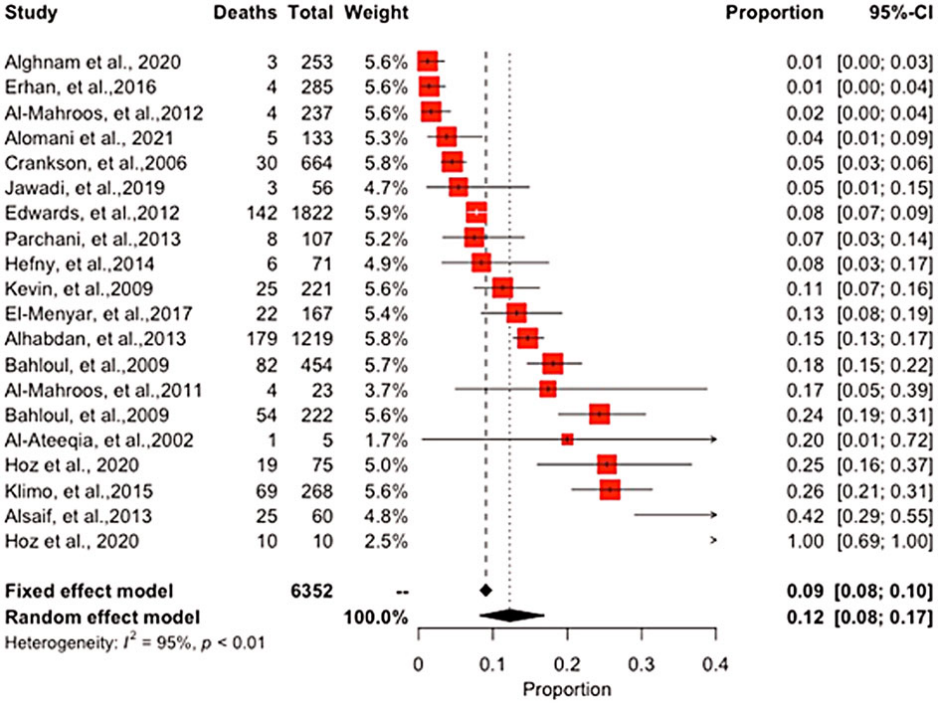
Meta-analysis of pediatric Traumatic Brain Injury.

Twenty studies reported on the pediatric death rate attributable to TBI—which varied widely across studies—ranging between 1.4% and 30%, yielding a grand total for the mortality rate of 16.3%. Leading causes included child abuse, MVAs, military injuries, and camel and other recreational injuries. In studies including multiple injury types, TBI was frequently reported as a leading cause of death. We report an overall pTBI pooled prevalence estimate of 12% (95% confidence interval [CI], 8–17; *I*^2^ = 95%; *p* < 0.01; Fig. 5).

**FIG. 6.**
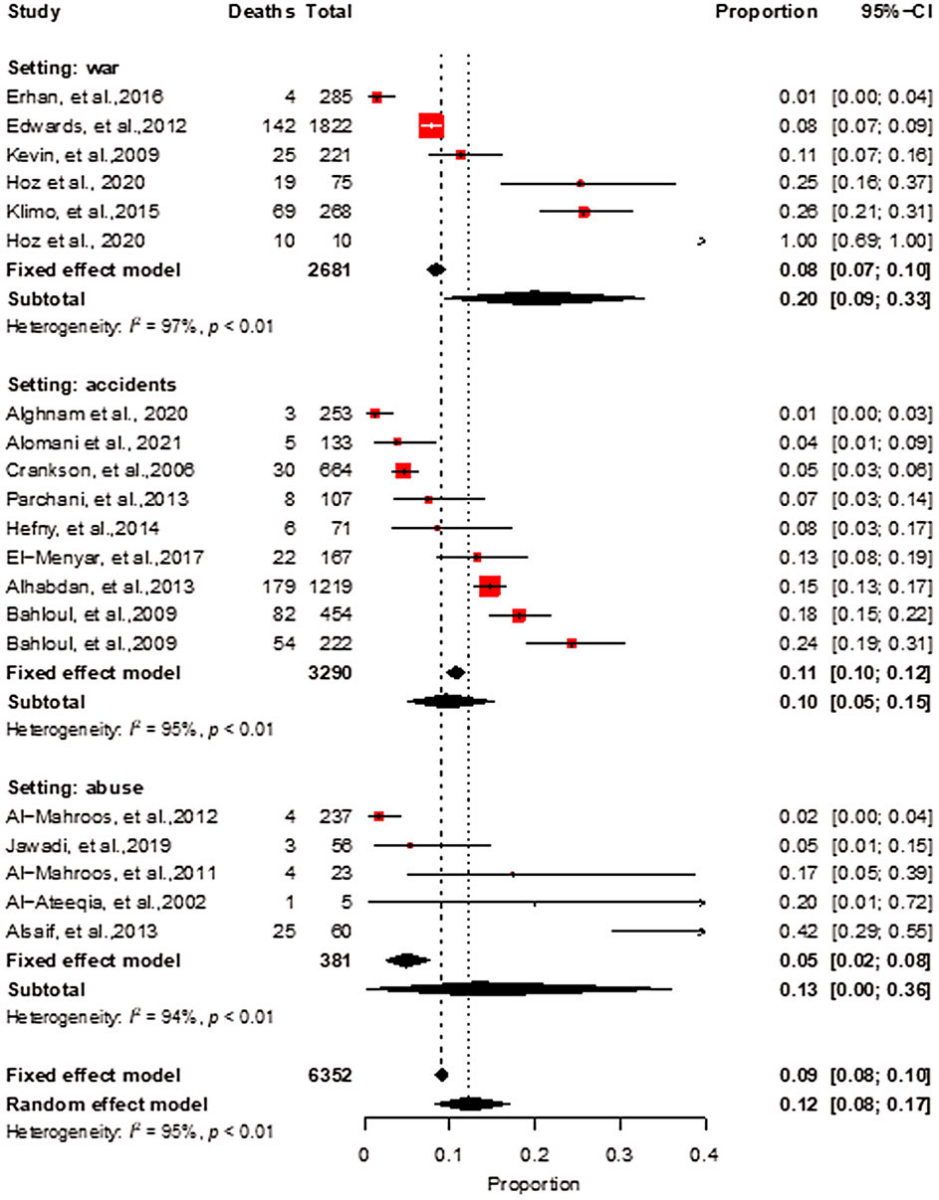
Meta-analysis of pediatric Traumatic Brain Injury with subgroup analysis by the setting of injury. Incidents include multiple causes, Motor Vehicle Accidents (MVA), camel-related injuries.

**FIG. 7.**
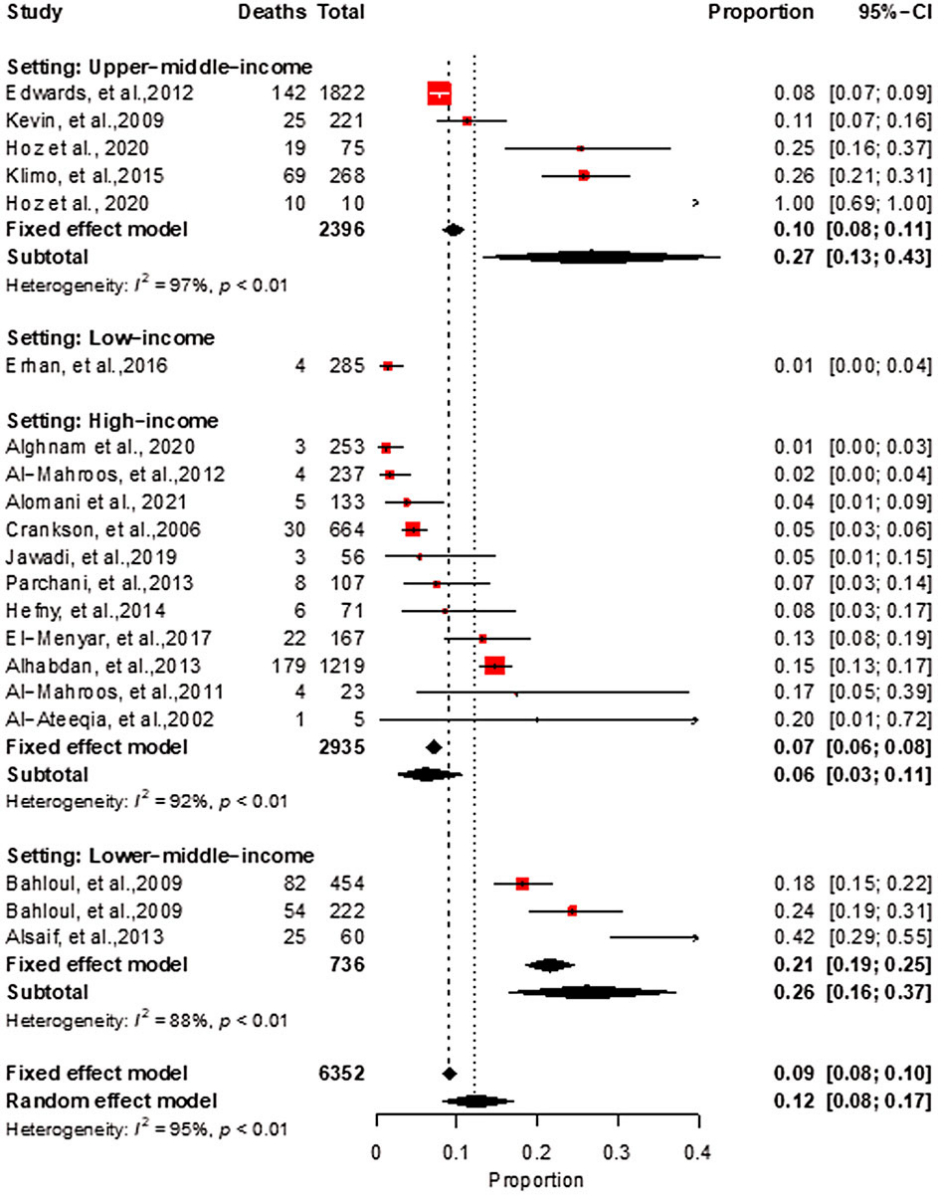
Meta-analysis of pediatric Traumatic Brain Injury with subgroup analysis by country income.

Subgroup analyses by the mechanism of TBI indicated a high heterogeneity as well, with pooled estimates of 20% for war injuries, 10% for road crashes, and 13% related to abuse (Fig. 6). When subgroup analysis is stratified by country income, a wide variation is noted with pooled estimates of 6% (95% CI, 3–11) in HICs, 26% (95% CI, 16–37) in LMICs, and 27% (95% CI, 13–43) in upper-middle-income countries (UMICs; Fig. 7). For this subgroup (i.e., by country income), we did not have enough studies from low-income countries; therefore, only one estimate is reported.

#### Severity

Nine studies reported on the injury severity of TBI cases.^8,10,18,24,26,28,30,31,41^ The most common TBIs were severe and were reported in seven studies.^8,10,24,26,28,30,41^ Three studies reported diagnosing moderate cases,^8,24,41^ whereas four reported mild TBIs.^8,18,24,41^ Eight of these studies reported on the severity distribution of TBI cases, using the modalities of classification such as ISS, PTS, the Pediatric Risk of Mortality, GCS, head AIS, and CT scans.^8,10,18,24,26,28,31,41^ In total, most classified TBI cases were reported as severe TBI (16.2% of the total sample size in the review), whereas mild (0.4%) and moderate (0.6%) cases were relatively rare (Table 5). The most adopted investigation tools for measuring TBI severity were GCS, AIS, and ISS.

**Table 5.** Distribution of Total TBI Cases by Mild, Moderate, Severe, or Unclassified Severity Across All Studies

	No. of cases	Percentage of cases
No. of mild cases	26	0.41
No. of moderate cases	38	0.60
No. of severe cases	1028	16.16
No. of unclassified cases	5269	82.83
Total sample	6361	100

TBI, traumatic brain injury.

#### Long-term outcomes

Eleven studies provided information on recovery and long-term outcomes among patients who survived.^17,18,20,21,24,30,31,33,34,38,42^ Five studies reported full recovery and return to baseline for the majority of patients.^20,21,33,34,42^ Three studies found severe disability in TBI pediatric patients (Glasgow Outcome Scale [GOS] = 3).^33,34,38^ Among these studies, Bahloul and colleagues found that whereas half of the patients (53%) had a good recovery, the other half sustained moderate severity and poor long-term outcomes, including epilepsy, motor weakness or paralysis, oculomotor problems, and sensorineural and language deficits, with few patients suffering from severe disability and coma.^21^ Hoz and colleagues reported moderate disability in 3.5% of cases (GOS = 4).^33^ Crankson reported neurological impairment in 9.3 of cases, including weakness, paralysis, cranial nerve palsies, and a vegetative state.^24^ One study found that the majority of TBI patients had a loss of urine and stool control (mean FIM = 2.7), on average, 9 months after TBI. Six studies reported observing patients in a vegetative state after the TBI.^20,21,24,33^

## Discussion

This meta-analysis is the first to gather literature across the Arab-speaking MENA region on pTBI and evaluate epidemiological trends, mechanisms, severity, sex distribution, and management. pTBI imposes a substantial human and economic burden on children, families, and healthcare systems. Accurate diagnosis and recognition of pTBI cases are critical for an accurate assessment of the true population burden. Though the knowledge surrounding the mechanisms and risk factors of pTBI is well established in many parts of the world, limited data exist in MENA countries. This review provides a deeper understanding of common risk factors and outcomes to gain perspective on the scope of the problem. Investigating the characteristics and extent of pTBIs among children will provide valuable insights for developing effective child injury-prevention strategies in the MENA region.

Our findings revealed an overall pediatric pTBI pooled prevalence estimate of 12%, which indicates a significant burden of TBI in children. However, the high heterogeneity observed (*I*^2^ = 95%) suggests that prevalence varies significantly across studies, and additional factors may contribute to the observed variation. Subgroup analysis by the mechanism of TBI indicated that war injuries had the highest prevalence of TBI at 20%, followed by abuse-related TBIs (13%) and road crashes (10%). This finding highlights the importance of identifying the cause of TBI to better understand the risk factors and provide targeted prevention and treatment strategies.

When examining country income, our analysis revealed a wide variation in pooled estimates, with the lowest prevalence of TBI (6%) in HICs and the highest prevalence in LMICs (26%) and UMICs (27%). This result suggests that socioeconomic factors may play a role in the incidence of TBI, and more research is needed to investigate this association.

The most commonly reported causes of pTBI encountered in the literature include falls (50.2%), struck by or against objects (24.8%), MVA (6.8%), assault (2.9%), and others (15.3%).^3^ Causes vary by age, given that falls, assault (e.g., shaken baby syndrome), and physical abuse are frequently observed among infants, toddlers, and pre-schoolers.^38^ Velocity injuries (including motor vehicle or bicycle crashes) and sports injuries are more often reported in elementary school children and adolescents.^38^ Males, especially those ages 0–9, have been reported to have 1.4 times the risk of sustaining moderate or severe pTBI relative to females of the same age group. Females, however, have a higher reported incidence of mild pTBI.^36^

The data showed that TBI affected both young adults and older adults, with males being the majority of cases in Lebanon. Nonetheless, TBI mortality, rehabilitation, and systemic injury data are rarely reported, with only three studies indicating rates for mild cases.^13^ Abusive TBI was found to be the most common cause of pediatric TBI in Saudi Arabia, whereas falls and MVAs were the leading causes in Oman and Qatar. Seatbelt compliance was associated with reduced morbidity and mortality in Qatar. However, it is noted that data on TBI mortality, rehabilitation, and systemic injury are often not reported, highlighting the need for further research in this area.

The results of this systematic review also revealed sex-based differences in the distribution of pTBI cases overall and by injury mechanism. Across studies, pTBI cases were predominantly males (75%). MVAs were the most prevalent mechanism, with males representing 86% cases. In the nine studies that revealed violence and assault as the mechanisms of injury, many abuse victims were males. However, it is interesting to note that homicide victims were more commonly females (53%) than males (46%). Among the seven studies related to the military, there were 65–75% more male pediatric cases than females. The remaining studies did not indicate differences between sexes.

Mechanisms of pTBI were also distinct across countries. The primary cause of pTBI will vary from caregiver abuse to fall injuries to motor vehicle crashes, and to military-related brain injuries, mostly prevalent in Iraq and Afghanistan.^10,25^ The higher number of military TBIs in these countries reflects the political unrest and enduring wars and conflicts they face. It is essential to understand each culture and the associated factors leading to TBI to implement interventions accordingly. Identifying the underlying causes could lead to significant improvements in TBI prevention, treatment, and continuum of care. However, causal mechanisms may exceed those indicated by research and should be further investigated.

pTBI remains the most consequential pediatric injury in terms of severity and outcomes. There were significant differences in the proportion of cases reported among pTBI severity levels. Of the 32 studies compiled in this review, the vast majority reported severe pTBI (16.25%) compared to mild (0.39%) and moderate (0.58%) severity. Several studies indicated TBI as the most common cause of mortality and disability.^3,4,8^ Commonly reported disabilities were meningeal hemorrhage and brain contusion, with the latter occurring proportionately higher among adolescents and teenagers. Teenagers (10–14 years) also had a higher mean ISS; however, GCS findings varied among studies. Median ventilation support days, ICU, and hospital length of stay were significantly prolonged in several studies.^3,5,39^

The study examined the tools used for TBI diagnosis, which may assist in developing clinical practices. Considering the implemented procedures, a series of tools were adopted by emergency medical services personnel to assess the severity of TBIs and consciousness sustained by patients. These tools included GCS, CT scan, x-ray, and surgical interventions. Improving the health outcomes of children requires determining the best clinical practices and minimizing variances in care. Further, undertaking more implementation-based research can ensure that effective therapies are applied to improve clinical outcomes.

### Implications and recommendations for future research and health professionals

This review recommends specifying the severity of TBIs upon diagnosis, which would allow future studies to analyze the results and understand health outcomes relative to injury severity. Accurate and comprehensive data collection by emergency physicians, neurosurgeons, and nursing staff is essential to further understanding pTBI in the MENA region. Data should include demographics, injury patterns and mechanisms, site and location of the injury, date of injury, and the length of time between the incident and provision of care. Training medical staff to use CT imaging, assess TBI severity, and provide operative and non-operative management is also crucial to evaluating and treating TBI patients. In addition, comorbidities and long-term outcomes should be further investigated.

Existing literature revealed the non-standardized documentation of available TBI studies in the MENA region. Aligning with international standards for a standardized documentation process is recommended to facilitate comparisons and effectively assess prevalence. There is also a need for coordinated efforts between government and communities. A multi-disciplinary approach to researching and implementing modern pTBI care is essential to prevention and management. Moreover, educating communities on pTBI, risk factors, and causes is a suitable method for raising awareness.

### Limitations

There were several limitations to this review. The heterogeneity of the included studies was a barrier to applying a concise meta-analysis. Further, the number of articles per country should be normalized to the population size to reduce bias. It was also difficult to verify whether the existing selected samples were representative of the country's population. Having representative samples is essential to assimilate the true prevalence of TBI within a population. Sampling variations further made it challenging to estimate the incidence rate and outcomes; this stems from non-standardized reporting and the absence of a universal system for reporting and classifying TBI cases. In addition, many of the included articles failed to mention multiple important variables, including the pTBI severity and SES of families and children. As a result, the reporting of results and analysis of pTBI consequences in the MENA region were affected. Other articles failed to mention the presented symptoms and complications associated with the pTBI. These two factors are highly essential given that they would alert the parents to escort their child to the emergency room for better management.

## Conclusion

This study has shed light on the various mechanisms of pTBI in the MENA region. Moreover, it has revealed how pTBI manifests in several forms, ranging from mild alterations of consciousness to severe morbidity and death. TBI among children is a significant health problem that affects the anatomy and physiology of the brain. However, developing primary healthcare services can prevent severe complications and mortality. The future recommendations are to gain a further understanding of pBI risk factors. Such insight can guide the development of strategies to reduce pTBI occurrence and improve health outcomes for communities within the MENA region.

## Supplementary Material

Supplemental data

## Supplementary Material

Appendix SA

## Abbreviations Used

AIS: Abbreviated Injury Scale
CI: confidence interval
CINHAL: Current Index to Nursing & Allied Health Literature
CT: computed tomography
DRS: Disability Rating Scale
FIM: Functional Independence Measurement
GCS: Glasgow Coma Scale
GOS: Glasgow Outcome Scale
HICs: high-income countries
ICU: intensive care unit
ISS: Injury Severity Score
KSA: Kingdom of Saudi Arabia
LMICs: lower-middle-income countries
MENA: Middle East and North Africa
MVAs: motor vehicle accidents
PRISMA: Preferred Reporting Items for Systematic Reviews and Meta-Analyses
pTBI: pediatric traumatic brain injury
PTS: Pediatric Trauma Score
SES: socioeconomic status
STROBE: Strengthening the Reporting of Observational Studies in Epidemiology
TBI: traumatic brain injury
UAE: United Arab Emirates
UMICs: upper-middle-income countries
WOS: Web of Science

## References

[1] Hyder AA, Wunderlich CA, Puvanachandra P, et al. The impact of traumatic brain injuries: a global perspective. NeuroRehabilitation 2007;22(5):341–353. 18162698

[2] Dewan MC, Rattani A, Gupta S, et al. Estimating the global incidence of traumatic brain injury. J Neurosurg 2018;130(4):1080–1097; doi: 10.3171/2017.10.JNS17352 29701556

[3] Centers for Disease Control and Prevention. Report to Congress on Traumatic Brain Injury Epidemiology and Rehabilitation: Recommendations for Addressing Critical Gaps. Centers for Disease Control and Prevention: Atlanta, GA; 2016.

[4] National Institute of Neurological Disorders and Stroke. Traumatic Brain Injury (TBI). Health Information page. National Institutes of Health: Bethesda, MD; 2023. Available from: https://www.ninds.nih.gov/health-information/disorders/traumatic-brain-injury-tbi [Last accessed: May 30, 2023].

[5] Sah P. Kids are more susceptible to brain injury, and concussion has implications beyond what we thought. 2016. Available from: [https://theconversation.com/kids-are-more-susceptible-to-brain-injury-and-concussion-has-implications-beyond-what-we-thought-64370] [Last accessed: May 29, 2023].

[6] Figaji AA. Anatomical and physiological differences between children and adults relevant to traumatic brain injury and the implications for clinical assessment and care. Front Neurol 2017;8:685; doi: 10.3389/fneur.2017.00685 29312119 PMC5735372

[7] Zaloshnja E, Miller T, Langlois JA, et al. Prevalence of long-term disability from traumatic brain injury in the civilian population of the United States, 2005. J Head Trauma Rehabil 2008;23(6):394–400; doi: 10.1097/01.HTR.0000341435.52004.ac 19033832

[8] El-Menyar A, Consunji R, Al-Thani H, et al. Pediatric traumatic brain injury: a 5-year descriptive study from the National Trauma Center in Qatar. World J Emerg Surg 2017;12(1):48; doi: 10.1186/s13017-017-0159-9 29151847 PMC5678808

[9] GBD 2016 Neurology Collaborators. Global, regional, and national burden of neurological disorders, 1990–2016: a systematic analysis for the Global Burden of Disease Study 2016. Lancet Neurol 2019;18(5):459–480; doi: 10.1016/S1474-4422(18)30499-X 30879893 PMC6459001

[10] Klimo P Jr, Ragel BT, Jones GM, et al. Severe pediatric head injury during the Iraq and Afghanistan conflicts. Neurosurgery 2015;77(1):1–7; discussion, 7; doi: 10.1227/NEU.0000000000000743 25812072

[11] Al-Hajj S, Hammoud Z, Colnaric J, et al.; the TBI Working Group. Characterization of traumatic brain injury research in the Middle East and North Africa Region: a systematic review. Neuroepidemiology 2021;55(1):20–31; doi: 10.1159/000511554 33567436

[12] El-Menyar A, Mekkodathil A, Al-Thani H, et al. Incidence, demographics, and outcome of traumatic brain injury in the Middle East: a systematic review. World Neurosurg 2017;107:6–21; doi: 10.1016/j.wneu.2017.07.070 28736357

[13] Abou-Abbass H, Bahmad H, Ghandour H, et al. Epidemiology and clinical characteristics of traumatic brain injury in Lebanon: a systematic review. Medicine (Baltimore) 2016;95(47):e5342; doi: 10.1097/MD.0000000000005342 27893670 PMC5134863

[14] Al-Adawi S, Al-Busaidi Z, Al-Adawi S, et al. Families coping with disability due to brain injury in Oman: attribution to belief in spirit infestation and ensorcellment. Sage Open 2012;2(3): doi: 10.1177/2158244012457400

[15] Al-Mahroos F, Al-Amer EA, Umesh NJ, et al. Pattern of skeletal injuries in physically abused children. Bahrain Med Bull 2011;33(2):67–71.

[16] Al-Ateeqi W, Shabani I, Abdulmalik A. Child abuse in Kuwait: problems in management. Med Princ Pract 2002;11(3):131–135; doi: 10.1159/000063241 12138294

[17] Al-Mahroos F, Al-Amer E. Child physical abuse in Bahrain: a 10-year study, 2000–2009. East Mediterr Health J 2012;18(6):579–585; doi: 10.26719/2012.18.6.579 22888614

[18] Alhabdan S, Zamakhshary M, Al Naimi M, et al. Epidemiology of traumatic head injury in children and adolescents in a major trauma center in Saudi Arabia: implications for injury prevention. Ann Saudi Med 2013;33(1):52–56; doi: 10.5144/0256-4947.2013.52 23458942 PMC6078585

[19] Alsaif D, Alsowayigh K, Alfaraidy M, et al. Child homicide in Cairo from 2006 to 2010: characteristics and trends. J Forensic Leg Med 2013;20(7):929–932; doi: 10.1016/j.jflm.2013.08.003 24112348

[20] Bahloul M, Hamida CB, Chelly H, et al. Severe head injury among children: prognostic factors and outcome. Injury 2009;40(5):535–540; doi: 10.1016/j.injury.2008.04.018 18703191

[21] Bahloul M, Chelly H, Gargouri R, et al. [Traumatic head injury in children in south Tunisia epidemiology, clinical manifestations and evolution. 454 cases.] [Article in French] Tunis Med 2009;87(1):28–37. 19522424

[22] Bener A, Al-Mulla FH, Al-Humoud SM, et al. Camel racing injuries among children. Clin J Sport Med 2005;15(5):290–293; doi: 10.1097/01.jsm.0000181440.33645.01 16162985

[23] Çelikel A, Karbeyaz K, Kararslan B, et al. Childhood casualties during civil war: Syrian experience. J Forensic Leg Med 2015;34:1–4; doi: 10.1016/j.jflm.2015.04.021 26165650

[24] Crankson SJ. Motor vehicle injuries in childhood: a hospital-based study in Saudi Arabia. Pediatr Surg Int 2006;22(8):641–645; doi: 10.1007/s00383-006-1715-7 16830162

[25] Creamer KM, Edwards MJ, Shields CH, et al. Pediatric wartime admissions to US military combat support hospitals in Afghanistan and Iraq: learning from the first 2,000 admissions. J Trauma Acute Care Surg 2009;67(4):762–768; doi: 10.1097/TA.0b013e31818b1e15 19820583

[26] Edwards MJ, Lustik M, Eichelberger MR, et al. Blast injury in children: an analysis from Afghanistan and Iraq, 2002–2010. J Trauma Acute Care Surg 2012;73(5):1278–1283; doi: 10.1097/TA.0b013e318270d3ee 23117384

[27] Er E, Çorbacıoğlu ŞK, Güler S, et al. Analyses of demographical and injury characteristics of adult and pediatric patients injured in Syrian civil war. Am J Emerg Med 2017;35(1):82–86; doi: 10.1016/j.ajem.2016.10.008 27771222

[28] Grivna M, Barss P, Stanculescu C, et al. Child and youth traffic-related injuries: use of a trauma registry to identify priorities for prevention in the United Arab Emirates. Traffic Inj Prev 2013;14(3):274–282; doi: 10.1080/15389588.2012.711498 23441946

[29] Grivna M, Eid HO, Abu-Zidan FM. Pediatric and youth traffic-collision injuries in Al Ain, United Arab Emirates: a prospective study. PLoS One 2013;8(7):e68636; doi: 10.1371/journal.pone.0068636 23861931 PMC3701680

[30] Al-Mahroos FT, Al-Amer EA, Al-Hashimi HA, et al. Abusive head trauma in children: the extent and clinical characteristics. Bahrain Med Bull 2011;33(4):174–174.

[31] Hawamdeh Z, Ibrahim A, Mezher A. Traumatic brain injury in the Gaza Strip: adults and children and their caregiver disability burden. Eur J Phys Rehabil Med 2011;47(2):193–201. 21150858

[32] Hefny AF, Eid HO, Abu-Zidan FM. Pedestrian injuries in the United Arab Emirates. Int J Inj Contr Saf Promot 2015;22(3):203–208; doi: 10.1080/17457300.2014.884143 24720810

[33] Hoz SS, Dolachee AA, Abdali HA, et al. An enemy hides in the ceiling; pediatric traumatic brain injury caused by metallic ceiling fan: case series and literature review. Br J Neurosurg 2019;33(3):360–364; doi: 10.1080/02688697.2019.1573312 30773933

[34] Jamous MA, Aziz HA, Al Kaisy F, et al. Conservative management of acute epidural hematoma in a pediatric age group. Pediatr Neurosurg 2009;45(3):181–184; doi: 10.1159/000218200 19440005

[35] Jawadi AH, Benmeakel M, Alkathiri M, et al. Characteristics of nonaccidental fractures in abused children in Riyadh, Saudi Arabia. Saudi J Med Med Sci 2019;7(1):9–15; doi: 10.4103/sjmms.sjmms_12_18 30787851 PMC6381845

[36] Kambal MA, Abou ME, Al Gadi I, et al. Managing traumatic brain injury in children: when do we need a computed tomography of the head? Sudan J Paediatr 2014;14(1):89–100. PMC4949923 27493396

[37] Khattab A, Othman Y. The outcome of severe traumatic brain injury in children in Qatar: six-year study. J Local Global Health Sci 2015;2015(Proceedings of the 24^th^ World International Traffic Medicine Association Congress, Qatar 2015):29; doi: 10.5339/jlghs.2015.itma.29

[38] Martin JE, Teff RJ, Spinella PC. Care of pediatric neurosurgical patients in Iraq in 2007: clinical and ethical experience of a field hospital. J Neurosurg Pediatr 2010;6(3):250–256; doi: 10.3171/2010.6.PEDS1031 20809709

[39] McGuigan R, Spinella PC, Beekley A, et al. Pediatric trauma: experience of a combat support hospital in Iraq. J Pediatr Surg 2007;42(1):207–210; doi: 10.1016/j.jpedsurg.2006.09.020 17208567

[40] Mehmood A, Agrawal P, Allen KA, et al. Childhood injuries in Oman: retrospective review of a multicentre trauma registry data. BMJ Paediatr Open 2018;2(1):e000310; doi: 10.1136/bmjpo-2018-000310 PMC6242029 30498792

[41] Nawaz A, Matta H, Hamchou M, et al. Camel-related injuries in the pediatric age group. J Pediatr Surg 2005;40(8):1248–1251; doi: 10.1016/j.jpedsurg.2005.05.006 16080927

[42] Nazer H, Daradkeh T, Mohamed S, et al. A diagnostic dilemma in Jordan: two child abuse case studies. Child Abuse Negl 1988;12(4):593–599; doi: 10.1016/0145-2134(88)90077-4 3233525

[43] Parchani A, El-Menyar A, Al-Thani H, et al. Recreational-related head injuries in Qatar. Brain Inj 2013;27(12):1450–1453; doi: 10.3109/02699052.2013.823664 23924056

[44] Alnasser M, AlSelaim N, Aldhukair S, et al. Patterns of pediatric trauma in Ramadan: an observational study. Ann Pediatr Surg 2012;8(1):9–11; doi: 10.1097/01.XPS.0000408270.86780.37

[45] Kemp AM, Dunstan F, Harrison S, et al. Patterns of skeletal fractures in child abuse: systematic review. BMJ 2008;337:a1518; doi: 10.1136/bmj.a1518 18832412 PMC2563260

[46] Kariyattil R, Muthukuttiparambil U. Traumatic acute brain herniation through the ear in a child: concealed compound fracture. Sultan Qaboos Univ Med J 2012;12(3):352–356; doi: 10.12816/0003150 22912929 PMC3413627

