# Supplementary material for: Pediatric Traumatic Brain Injury in the Middle East and North Africa Region: A Systematic Review and Meta-Analysis to Assess Characteristics, Mechanisms, and Risk Factors
Source: Neurotrauma Rep. 2023 Oct 17;4(1):693–714. doi: 10.1089/neur.2023.0007 (PMC10615069; doi:10.1089/neur.2023.0007)
Supplement: Supplemental data [file Suppl_AppendixSA.docx]

**Supplementary Material A**

Search Strategy

(See the full search strategy in Supplementary Material A).

***SCOPUS- 1,230***

( ( TITLE-ABS-KEY ( ( ( ( ( child*  OR  female*  OR  male*  OR  m*n  OR  wom*n  OR  adult*  OR  boy*  OR  girl*  OR  adolescent*  OR  old  OR  elderly  OR  past )  PRE/0  trauma* )  OR  traumatic  OR  war*  OR  battle*  OR  combat*  OR  post-traumatic  OR  posttraumatic  OR  combat-related  OR  combat-associated  OR  combat-induced  OR  post-combat )  AND  ( mental*  OR  psychiat*  OR  psycholog*  OR  psychic  OR  ideation*  OR  idea*  OR  thought*  OR  stress  OR  stressful  OR  stresses ) ) ) )  OR  ( TITLE-ABS-KEY ( ( illness  AND  ( frighten*  OR  fight*  OR  war  OR  combat*  OR  battle* ) ) ) )  OR  ( TITLE-ABS-KEY ( ( psycholog*  W/3  ( ( witness*  OR  experienc* ) ) ) ) )  OR  ( TITLE-ABS-KEY ( ( psycholog*  W/2  ( scar*  OR  suffering ) ) ) )  OR  ( TITLE-ABS-KEY ( ( recurren*  W/2  ( thought*  OR  image* ) ) ) )  OR  ( TITLE-ABS-KEY ( ( ( survivor*  OR  war*  OR  hysterical )  PRE/0  guilt ) ) )  OR  ( TITLE-ABS-KEY ( ( ( war  OR  hysterical )  PRE/0  neuro* ) ) )  OR  ( TITLE-ABS-KEY ( ( ( shell  OR  mental* )  W/1  shock* ) ) )  OR  ( TITLE-ABS-KEY ( ( ( battle*  OR  war*  OR  ( conflict  PRE/0  ( zone  OR  area ) )  OR  combat  OR  combat-related  OR  combat-induced  OR  combat-associated )  AND  ( psychiat*  OR  psycholog*  OR  mental*  OR  stress  OR  stressful*  OR  disorder*  OR  fatigue*  OR  neuros*s ) ) ) )  OR  ( TITLE-ABS-KEY ( ( stress*  W/2  ( scale*  OR  score* ) ) ) )  OR  ( TITLE-ABS-KEY ( assault*  OR  ( ptss  OR  ptsd  OR  ( railway  PRE/0  spine )  OR  ( ( post-trauma*  OR  posttrauma*  OR  delayed )  PRE/0  stress  PRE/0  ( disorder*  OR  syndrome*  OR  symptom* ) ) )  OR  ( ( adverse  PRE/0  childhood  PRE/0  experience* )  OR  ( ( domestic  OR  family  OR  gun  OR  gender-based  OR  work*  OR  expos* )  W/2  violence )  OR  ( ( child*  OR  minor*  OR  kid*  OR  physical*  OR  elder*  OR  spouse*  OR  partner* )  W/2  abus* )  OR  rape  OR  raping  OR  raped  OR  terroris*  OR  bioterroris*  OR  bio-terroris*  OR  ( mass  PRE/0  casualt*  PRE/0  incidence )  OR  torture  OR  ( dowry  PRE/0  death* ) ) ) ) )

AND

  ( TITLE-ABS-KEY ( ( middle  W/2  east* )  OR  ( north*  W/2  africa* )  OR  ( east*  W/2  mediterranean )  OR  lebanon  OR  lebanese  OR  algeria*  OR  bahrain*  OR  egypt*  OR  iraq*  OR  jordan*  OR  kuwait*  OR  luban*  OR  lobnan*  OR  leban*  OR  libanaise  OR  yemen*  OR  dubai  OR  ( abu  W/2  dhabi )  OR  aden  OR  sanaa  OR  uae  OR  emirat*  OR  libya*  OR  morocco  OR  moroccan*  OR  ifni  OR  "trucial state"  OR  oman*  OR  muscat  OR  palestin*  OR  ghaza  OR  ghazza  OR  gaza  OR  ( west*  W/2  bank )  OR  qatar*  OR  katar*  OR  quatar*  OR  saudi*  OR  ksa  OR  syria*  OR  tunis*  OR  levant  OR  mena  OR  emro  OR  orient  OR  arabs  OR  arab  OR  arabia ) )

AND

  ( ( TITLE-ABS-KEY ( ( ( brain*  OR  encephalon*  OR  cranium  OR  cranio*  OR  cranial  OR  intracrani*  OR  intra-crani*  OR  skull*  OR  cerebral  OR  cerebell*  OR  head  OR  ventric*  OR  hemispher*  OR  pontine  OR  putamin*  OR  dura*  OR  subdura*  OR  sub-dura*  OR  ( ( supra  OR  extra )  W/1  dura* )  OR  supradura*  OR  epidura*  OR  epi-dura*  OR  arachnoid*  OR  sub-arachnoid*  OR  subarachnoid*  OR  ( intra  W/1  arachnoid* ) )  W/2  ( injur*  OR  trauma*  OR  concussion*  OR  post-concussion*  OR  h*emorrhag*  OR  h*ematom*  OR  bleed*  OR  penetrat*  OR  ( non  W/1  penetrat* )  OR  edema*  OR  oedema*  OR  fracture*  OR  aneurysm*  OR  pressur*  OR  lesion*  OR  swell*  OR  contusion* ) ) ) )  OR  ( TITLE-ABS-KEY ( ( neurosurg*  OR  neuro-surg*  OR  craniotom*  OR  craniectom*  OR  trepanation*  OR  trepanning*  OR  trephination*  OR  trephining* ) ) )  OR  ( TITLE-ABS-KEY ( pneumocephal*  OR  pneumo-cephal*  OR  a*rocele*  OR  pneumocyst*  OR  pneumo-cyst*  OR  ( ( csf  OR  cerebrospinal )  W/2  ( leak*  OR  otorrhea*  OR  rhinorrhea* ) )  OR  co*ntrecoup*  OR  ( co*ntre  W/2  coup* ) ) )  OR  ( TITLE-ABS-KEY ( ( ( force  OR  brain  OR  skull  OR  blunt  OR  craniocervical  OR  ( nervous  PRE/0  system* )  OR  craniocerebral  OR  forehead  OR  ( ( parietal  OR  temporal  OR  frontal )  PRE/0  region )  OR  occipital )  W/2  ( blunt*  OR  fractur*  OR  damag*  OR  defect*  OR  lesion*  OR  blast*  OR  trauma*  OR  injur*  OR  concussion*  OR  head* ) ) ) )  OR  ( TITLE-ABS-KEY ( ( ibt  OR  bit  OR  cti  OR  bits  OR  tbi*  OR  mtbi*  OR  neurotmes*s  OR  concussion*  OR  ( commotio  PRE/0  cerebri )  OR  contusio*  OR  ( post  W/0  ( trauma*  OR  concussive )  W/2  encephalopath* )  OR  ( brain  PRE/0  laceration* ) ) ) ) )

***Medline/Ovid- 970***

Database: Ovid MEDLINE(R) and Epub Ahead of Print, In-Process & Other Non-Indexed Citations and Daily <1946 to May 08, 2020>

Search Strategy:

--------------------------------------------------------------------------------

1 ((((child* or female? or male? or m?n or wom?n or adult? or boy? or girl? or adolescent? or old or elderly or past) adj trauma?) or traumatic or War* or Battle* or Combat? or Post-Traumatic or posttraumatic or combat-related or combat-associated or combat-induced or post-combat) and (mental* or psychiat* or psycholog* or psychic or ideation? or idea? or thought? or stress or stressful or stresses)).mp. (147058)

2 (illness and (frighten* or fight* or war or combat* or battle*)).mp. (3760)

3 (psycholog* adj4 ((witness* or experienc*) adj shock*)).mp. (1)

4 (psycholog* adj3 (scar? or suffering)).mp. (695)

5 (recurren* adj3 (thought? or image?)).mp. (557)

6 ((survivor? or war* or hysterical) adj guilt).mp. (81)

7 ((war or hysterical) adj neuro*).mp. (248)

8 ((shell or mental*) adj2 shock*).mp. (168)

9 ((battle* or war* or (conflict adj (zone or area)) or combat or combat-related or combat-induced or combat-associated) and (psychiat* or psycholog* or mental* or stress or stressful* or disorder* or fatigue* or neuros?s)).mp. (103573)

10 (Stress* adj3 (scale? or score?)).mp. (9215)

11 assault?.mp. (13131)

12 (Ptss or ptsd or (railway adj spine) or ((post-trauma* or posttrauma* or delayed) adj stress adj (disorder? or syndrome? or symptom?))).mp. (35349)

13 Stress Disorders, Post-Traumatic/ or exp violence/ (119920)

14 ((Adverse adj Childhood adj Experience*) or ((Domestic or family or gun or gender-based or work* or expos*) adj3 Violence) or ((child* or minor* or kid? or physical* or elder* or spouse* or partner?) adj3 abus*) or rape or raping or raped or terroris* or bioterroris* or bio-terroris* or (mass adj casualt* adj incidence) or torture or (Dowry adj Death?)).mp. (91150)

15 africa, northern/ or algeria/ or egypt/ or libya/ or morocco/ or tunisia/ or middle east/ or bahrain/ or iraq/ or jordan/ or kuwait/ or lebanon/ or oman/ or qatar/ or saudi arabia/ or syria/ or united arab emirates/ or yemen/ or arabs/ or (algeria* or bahrain* or egypt* or iraq* or jordan* or kuwait* or kuweit* or leban* or liban* or lubnan* or lobnan* or yemen* or aden or sanaa or UAE or Emirat* or (abu adj dhabi) or dubai or libya* or morocco or moroccan* or oman* or muscat or palestin* or ghazza or ghaza or gaza or (west* adj2 bank) or qatar* or katar* or quatar* or saudi* or KSA or Syria* or tunis* or ((east* or north*) adj2 africa*) or ifni or (trucial adj state*) or MENA or EMRO or ((middle or near) adj2 east*) or (east* adj2 mediterranean) or orient or arabs or arab or arabia or levant).mp. (173440)

16 ((brain* or encephalon* or cranium or cranio* or cranial or intracrani* or intra-crani* or skull* or cerebral or cerebell* or head or ventric* or hemispher* or pontine or putamin* or dura* or subdura* or sub-dura* or ((supra or extra) adj2 dura*) or supradura* or epidura* or epi-dura* or arachnoid* or sub-arachnoid* or subarachnoid* or (intra adj2 arachnoid*)) adj3 (injur* or trauma* or concussion* or post-concussion* or h?emorrhag* or h?ematom* or bleed* or penetrat* or (non adj2 penetrat*) or edema* or oedema* or fracture* or aneurysm* or pressur* or lesion* or swell* or contusion*)).mp. (371281)

17 Neurosurgery/ or exp craniotomy/ (30006)

18 (neurosurg* or neuro-surg* or craniotom* or craniectom* or trepanation* or trepanning* or trephination* or trephining*).mp. (98457)

19 brain injury, chronic/ or brain edema/ or exp brain concussion/ or exp brain injuries, traumatic/ or exp hematoma, epidural, cranial/ or exp hematoma, subdural/ or subarachnoid hemorrhage, traumatic/ or exp pituitary apoplexy/ or craniocerebral trauma/ or exp intracranial hemorrhages/ or exp intracranial hypertension/ or exp trauma, nervous system/ (278151)

20 (pneumocephal* or pneumo-cephal* or a?rocele* or pneumocyst* or pneumo-cyst* or ((CSF or cerebrospinal) adj3 (leak* or otorrhea* or rhinorrhea*)) or co?ntrecoup* or (co?ntre adj3 coup*)).mp. (25751)

21 ((force or brain or skull or blunt or craniocervical or (nervous adj system?) or craniocerebral or forehead or ((parietal or temporal or frontal) adj region) or occipital) adj3 (blunt? or fractur* or damag* or defect? or lesion? or blast? or trauma* or injur* or concussion? or head?)).mp. (222272)

22 (ibt or bit or cti or bits or tbi? or mtbi? or neurotmes?s or concussion? or (commotio adj cerebri) or contusio* or (post adj (trauma* or concussive) adj3 encephalopath*) or (brain adj laceration?)).mp. (65403)

23 (or/1-14) and 15 and (or/16-22) (970)

***************************

***Global Index Medicus- 133&33***

- IMEMR (Eastern Mediterranean)[(remover)](javascript:remove_filter('db_imemr'))
- AIM (Africa) [(remover)](javascript:remove_filter('db_aim'))

AND Post-stress traumatic disorder

133 results

Post-stress traumatic disorder AND brain trauma

33 results

***Cochrane- 70***

Search Name: PTSD & TBI & MENA

Date Run: 12/05/2020 02:15:06

Comment:

ID Search Hits

#1 MeSH descriptor: [Neurosurgery] this term only 73

#2 MeSH descriptor: [Craniotomy] explode all trees 428

#3 MeSH descriptor: [Brain Injury, Chronic] this term only 34

#4 MeSH descriptor: [Brain Edema] this term only 196

#5 MeSH descriptor: [Brain Concussion] explode all trees 325

#6 MeSH descriptor: [Brain Injuries, Traumatic] explode all trees 684

#7 MeSH descriptor: [Hematoma, Epidural, Cranial] explode all trees 12

#8 MeSH descriptor: [Hematoma, Subdural] explode all trees 113

#9 MeSH descriptor: [Subarachnoid Hemorrhage, Traumatic] this term only 2

#10 MeSH descriptor: [Pituitary Apoplexy] explode all trees 1

#11 MeSH descriptor: [Intracranial Hemorrhages] explode all trees 1909

#12 MeSH descriptor: [Intracranial Hypertension] explode all trees 191

#13 MeSH descriptor: [Trauma, Nervous System] explode all trees 5082

#14 MeSH descriptor: [Craniocerebral Trauma] this term only 330

#15 ((brain* or encephalon* or cranium or cranio* or cranial or intracrani* or intra-crani* or skull* or cerebral or cerebell* or head or ventric* or hemispher* or pontine or putamin* or dura* or subdura* or sub-dura* or ((supra or extra) NEAR/2 dura*) or supradura* or epidura* or epi-dura* or arachnoid* or sub-arachnoid* or subarachnoid* or (intra NEAR/2 arachnoid*)) NEAR/3 (injur* or trauma* or concussion* or post-concussion* or hemorrhag* or haemorrhag* or hematom* or haematom* or bleed* or penetrat* or (non NEAR/2 penetrat*) or edema* or oedema* or fracture* or aneurysm* or pressur* or lesion* or swell* or contusion*)):ti,ab,kw 25015

#16 (neurosurg* or neuro-surg* or craniotom* or craniectom* or trepanation* or trepanning* or trephination* or trephining*):ti,ab,kw 4129

#17 (pneumocephal* or pneumo-cephal* or a?rocele* or pneumocyst* or pneumo-cyst* or ((CSF or cerebrospinal) NEAR/3 (leak* or otorrhea* or rhinorrhea*)) or contrecoup* or countrecoup* or (co?ntre NEAR/3 coup*)):ti,ab,kw 926

#18 ((force or brain or skull or blunt or craniocervical or (nervous NEXT system?) or craniocerebral or forehead or ((parietal or temporal or frontal) NEXT region) or occipital) NEAR/3 (blunt? or fractur* or damag* or defect? or lesion? or blast? or trauma* or injur* or concussion? or head?)) 11587

#19 ((ibt or bit or cti or bits or tbi? or mtbi? or neurotmes?s or concussion? or (commotio NEXT cerebri) or contusio* or ((post NEXT (trauma* or concussive)) NEAR/3 encephalopath*) or (brain NEXT laceration?))):ti,ab,kw 5977

#20 #1 or #2 #3 or #4 or #5 or #6 or #7 or #8 or #9 or #10 OR #11 OR #12 OR #13 OR #14 OR #15 OR #16 OR #17 OR #18 OR #19 37730

#21 MeSH descriptor: [Africa, Northern] explode all trees 478

#22 MeSH descriptor: [Middle East] this term only 41

#23 MeSH descriptor: [Bahrain] this term only 8

#24 MeSH descriptor: [Libya] this term only 6

#25 MeSH descriptor: [Iraq] this term only 48

#26 MeSH descriptor: [Jordan] this term only 70

#27 MeSH descriptor: [Kuwait] this term only 33

#28 MeSH descriptor: [Lebanon] this term only 57

#29 MeSH descriptor: [Oman] this term only 17

#30 MeSH descriptor: [Qatar] this term only 19

#31 MeSH descriptor: [Saudi Arabia] this term only 162

#32 MeSH descriptor: [Syria] this term only 25

#33 MeSH descriptor: [United Arab Emirates] this term only 22

#34 MeSH descriptor: [Yemen] this term only 5

#35 (algeria* or bahrain* or egypt* or iraq* or jordan* or kuwait* or leban* or liban* or lubnan* or lobnan* or yemen* or aden or sanaa or UAE or Emirat* or (abu NEAR/1 dhabi) or libya* or morocco* or moroccan* or oman* or muscat or palestin* or ghazza or ghaza or gaza or (west NEAR/1 bank) or qatar* or katar* or quatar* or saudi* or KSA or Syria* or tunisia* or (north* NEAR/2 africa*) or MENA or (middle NEAR/2 east*) or (east* NEAR/2 mediterranean*) or orient or arabs or arab or levant or arabia):ti,ab,kw 5965

#36 #21 or #22 or #23 or #24 or #25 or #26 or #27 or #28 or #29 or #30 or #31 or #32 or #33 or #34 or #35 5965

#37 (((((child* or female? or male? or m?n or wom?n or adult? or boy? or girl? or adolescent? or old or elderly or past) NEXT trauma?) or traumatic or War* or Battle* or Combat? or Post-Traumatic or posttraumatic or combat-related or combat-associated or combat-induced or post-combat) and (mental* or psychiat* or psycholog* or psychic or ideation? or idea? or thought? or stress or stressful or stresses))):ti,ab,kw 15080

#38 ((illness and (frighten* or fight* or war or combat* or battle*))):ti,ab,kw 280

#39 ((psycholog* NEAR/4 ((witness* or experienc*) adj shock*))):ti,ab,kw 758

#40 ((psycholog* NEAR/3 (scar? or suffering))):ti,ab,kw 58

#41 ((recurren* NEAR/3 (thought? or image?))):ti,ab,kw 62

#42 (((survivor? or war* or hysterical) NEXT guilt)):ti,ab,kw 3

#43 (((war or hysterical) NEXT neuro*)):ti,ab,kw 2

#44 (((shell or mental*) NEAR/2 shock*)):ti,ab,kw 5

#45 (((battle* or war* or (conflict NEXT (zone or area)) or combat or combat-related or combat-induced or combat-associated) and (psychiat* or psycholog* or mental* or stress or stressful* or disorder* or fatigue* or neuros?s))):ti,ab,kw 12493

#46 ((Stress* NEAR/3 (scale? or score?))):ti,ab,kw 3132

#47 (((battle* or war* or (conflict NEXT (zone? or area?))) NEXT fatigu*)):ti,ab,kw 19

#48 (assault? or (Ptss or ptsd or (railway NEXT spine) or ((post-trauma* or posttrauma* or delayed) NEXT stress NEXT (disorder? or syndrome? or symptom?)))):ti,ab,kw 5772

#49 (((Adverse NEXT Childhood NEXT Experience*) or ((Domestic or family or gun or gender-based or work* or expos*) NEAR/3 Violence) or ((child* or minor* or kid? or physical* or elder* or spouse* or partner?) NEAR/3 abus*) or rape or raping or raped or terroris* or bioterroris* or bio-terroris* or (mass NEXT casualt* NEXT incidence) or torture or (Dowry NEXT Death?))):ti,ab,kw 2753

#50 MeSH descriptor: [Stress Disorders, Post-Traumatic] this term only 2494

#51 MeSH descriptor: [Violence] explode all trees 1555

#52 #37 OR #38 OR #39 OR #40 OR #41 OR #42 OR #43 OR #44 OR #45 OR #46 OR #47 OR #48 OR #49 OR #50 OR #51 25810

#53 #20 AND #36 AND #52 70

***EMBASE- 1074***

| **No.** | **Query** | **Results** | **Date** |
| --- | --- | --- | --- |
| #36 | #20 AND #21 AND #35 | 1074 | 12-May-20 |
| #35 | #22 OR #23 OR #24 OR #25 OR #26 OR #27 OR #28 OR #29 OR #30 OR #31 OR #32 OR #33 OR #34 | 373245 | 12-May-20 |
| #34 | ((adverse NEXT/1 childhood NEXT/1 experience*):ti,ab,kw) OR (((domestic OR family OR gun OR 'gender based' OR work* OR expos*) NEAR/3 violence):ti,ab,kw) OR (((child* OR minor* OR kid$ OR physical* OR elder* OR spouse* OR partner$) NEAR/3 abus*):ti,ab,kw) OR rape:ti,ab,kw OR raping:ti,ab,kw OR raped:ti,ab,kw OR terroris*:ti,ab,kw OR bioterroris*:ti,ab,kw OR 'bio terroris*':ti,ab,kw OR ((mass NEXT/1 casualt* NEXT/1 incidence):ti,ab,kw) OR torture:ti,ab,kw OR ((dowry NEXT/1 death$):ti,ab,kw) | 71880 | 12-May-20 |
| #33 | assault$:ti,ab,kw OR ptss:ti,ab,kw OR ptsd:ti,ab,kw OR ((railway NEXT/1 spine):ti,ab,kw) OR ((('post trauma*' OR posttrauma* OR delayed) NEXT/1 stress NEXT/1 (disorder$ OR syndrome$ OR symptom$)):ti,ab,kw) | 63450 | 12-May-20 |
| #32 | (stress* NEAR/3 (scale$ OR score$)):ti,ab,kw | 13887 | 12-May-20 |
| #31 | (battle*:ti,ab,kw OR war*:ti,ab,kw OR ((conflict NEXT/1 (zone OR area)):ti,ab,kw) OR combat:ti,ab,kw OR 'combat related':ti,ab,kw OR 'combat induced':ti,ab,kw OR 'combat associated':ti,ab,kw) AND (psychiat*:ti,ab,kw OR psycholog*:ti,ab,kw OR mental*:ti,ab,kw OR stress:ti,ab,kw OR stressful*:ti,ab,kw OR disorder*:ti,ab,kw OR fatigue*:ti,ab,kw OR neuros$s:ti,ab,kw) | 99601 | 12-May-20 |
| #30 | ((shell OR mental*) NEAR/2 shock*):ti,ab,kw | 195 | 12-May-20 |
| #29 | ((war OR hysterical) NEXT/1 neuro*):ti,ab,kw | 301 | 12-May-20 |
| #28 | ((survivor$ OR war* OR hysterical) NEXT/1 guilt):ti,ab,kw | 98 | 12-May-20 |
| #27 | (recurren* NEAR/3 (thought$ OR image$)):ti,ab,kw | 953 | 12-May-20 |
| #26 | (psycholog* NEAR/3 (scar$ OR suffering)):ti,ab,kw | 1095 | 12-May-20 |
| #25 | (psycholog* NEAR/4 (witness* OR experienc*)):ti,ab,kw | 7655 | 12-May-20 |
| #24 | illness:ti,ab,kw AND (frighten*:ti,ab,kw OR fight*:ti,ab,kw OR war:ti,ab,kw OR combat*:ti,ab,kw OR battle*:ti,ab,kw) | 3505 | 12-May-20 |
| #23 | ((((child* OR female$ OR male$ OR m$n OR wom$n OR adult$ OR boy$ OR girl$ OR adolescent$ OR old OR elderly OR past) NEXT/1 trauma$):ti,ab,kw) OR traumatic:ti,ab,kw OR war*:ti,ab,kw OR battle*:ti,ab,kw OR combat$:ti,ab,kw OR 'post traumatic':ti,ab,kw OR posttraumatic:ti,ab,kw OR 'combat related':ti,ab,kw OR 'combat associated':ti,ab,kw OR 'combat induced':ti,ab,kw OR 'post combat':ti,ab,kw) AND (mental*:ti,ab,kw OR psychiat*:ti,ab,kw OR psycholog*:ti,ab,kw OR psychic:ti,ab,kw OR ideation$:ti,ab,kw OR idea$:ti,ab,kw OR thought$:ti,ab,kw OR stress:ti,ab,kw OR stressful:ti,ab,kw OR stresses:ti,ab,kw) | 142288 | 12-May-20 |
| #22 | 'posttraumatic stress disorder'/exp OR 'violence'/exp | 198178 | 12-May-20 |
| #21 | 'middle east'/de OR 'north africa'/exp OR 'lebanon'/exp OR 'syrian arab republic'/exp OR 'egypt'/exp OR 'algeria'/exp OR 'iraq'/exp OR 'bahrain'/exp OR 'jordan'/exp OR 'kuwait'/exp OR 'libya'/exp OR 'morocco'/exp OR 'tunisia'/exp OR 'oman'/exp OR 'qatar'/exp OR 'saudi arabia'/exp OR 'united arab emirates'/exp OR 'yemen'/exp OR 'palestine'/exp OR 'arab'/exp OR algeria*:ti,ab,kw OR bahrain*:ti,ab,kw OR egypt*:ti,ab,kw OR iraq*:ti,ab,kw OR jordan*:ti,ab,kw OR kuwait*:ti,ab,kw OR kuweit*:ti,ab,kw OR lebanon:ti,ab,kw OR lebanese:ti,ab,kw OR libanaise:ti,ab,kw OR yemen*:ti,ab,kw OR aden:ti,ab,kw OR sanaa:ti,ab,kw OR uae:ti,ab,kw OR emirat*:ti,ab,kw OR ((abu NEXT/1 dhabi):ti,ab,kw) OR dubai:ti,ab,kw OR libya*:ti,ab,kw OR morocco:ti,ab,kw OR moroccan*:ti,ab,kw OR ifni:ti,ab,kw OR ((trucial NEAR/2 state*):ti,ab,kw) OR oman*:ti,ab,kw OR muscat:ti,ab,kw OR palestin*:ti,ab,kw OR gaza:ti,ab,kw OR ((west NEXT/1 bank):ti,ab,kw) OR qatar*:ti,ab,kw OR katar*:ti,ab,kw OR quatar*:ti,ab,kw OR saudi*:ti,ab,kw OR ksa:ti,ab,kw OR syria*:ti,ab,kw OR tunis*:ti,ab,kw OR (((east* OR north*) NEAR/2 africa*):ti,ab,kw) OR mena:ti,ab,kw OR emro:ti,ab,kw OR (((middle OR near) NEAR/2 east*):ti,ab,kw) OR ((east* NEXT/2 mediterranean):ti,ab,kw) OR orient:ti,ab,kw OR arabs:ti,ab,kw OR arab:ti,ab,kw OR arabia:ti,ab,kw OR levant:ti,ab,kw | 212264 | 12-May-20 |
| #20 | #1 OR #2 OR #3 OR #4 OR #5 OR #6 OR #7 OR #8 OR #9 OR #10 OR #11 OR #12 OR #13 OR #14 OR #15 OR #16 OR #17 OR #18 OR #19 | 948556 | 12-May-20 |
| #19 | 'neurosurgery'/de OR 'craniotomy'/de OR 'brain injury'/exp OR 'head injury'/de OR 'brain edema'/de OR 'epidural hematoma'/de OR 'subdural hematoma'/de OR 'subarachnoid hemorrhage'/exp OR 'hypophysis apoplexy'/de OR 'brain hemorrhage'/exp OR 'intracranial hypertension'/exp OR 'nervous system injury'/exp | 619166 | 12-May-20 |
| #18 | neurosurg*:ti,ab,kw OR 'neuro surg*':ti,ab,kw OR craniotom*:ti,ab,kw OR craniectom*:ti,ab,kw OR trepanation*:ti,ab,kw OR trepanning*:ti,ab,kw OR trephination*:ti,ab,kw OR trephining*:ti,ab,kw | 95500 | 12-May-20 |
| #17 | ibt:ti,ab,kw OR bit:ti,ab,kw OR cti:ti,ab,kw OR bits:ti,ab,kw OR tbi$:ti,ab,kw OR mtbi$:ti,ab,kw OR neurotmes$s:ti,ab,kw OR concussion$:ti,ab,kw OR ((commotio NEXT/1 cerebri):ti,ab,kw) OR contusio*:ti,ab,kw OR ((post NEXT/1 (trauma* OR concussive) NEAR/3 encephalopath*):ti,ab,kw) OR ((brain NEXT/1 laceration$):ti,ab,kw) | 86234 | 12-May-20 |
| #16 | ((brain* OR encephalon* OR cranium OR cranio* OR cranial OR intracrani* OR 'intra crani*' OR skull* OR cerebral OR cerebell* OR head OR ventric* OR hemispher* OR pontine OR putamin* OR dura* OR subdura* OR 'sub dura*' OR supradura* OR epidura* OR 'epi dura*' OR arachnoid* OR 'sub arachnoid*' OR subarachnoid* OR 'intra arachnoid*' OR 'supra dura*' OR 'extra dura*') NEAR/3 (injur* OR trauma* OR concussion* OR 'post concussion*' OR h$emorrhag* OR h$ematom* OR bleed* OR penetrat* OR edema* OR oedema* OR fracture* OR aneurysm* OR pressur* OR lesion* OR swell* OR contusion* OR 'non penetrat*')):ti,ab,kw | 417563 | 12-May-20 |
| #15 | ((force OR brain OR skull OR blunt OR craniocervical OR 'nervous-system$' OR craniocerebral OR forehead OR 'parietal region' OR 'temporal region' OR 'frontal region' OR occipital) NEAR/3 head$):ti,ab,kw | 6064 | 12-May-20 |
| #14 | ((force OR brain OR skull OR blunt OR craniocervical OR 'nervous-system$' OR craniocerebral OR forehead OR 'parietal region' OR 'temporal region' OR 'frontal region' OR occipital) NEAR/3 concussion$):ti,ab,kw | 1324 | 12-May-20 |
| #13 | ((force OR brain OR skull OR blunt OR craniocervical OR 'nervous-system$' OR craniocerebral OR forehead OR 'parietal region' OR 'temporal region' OR 'frontal region' OR occipital) NEAR/3 injur*):ti,ab,kw | 120791 | 12-May-20 |
| #12 | ((force OR brain OR skull OR blunt OR craniocervical OR 'nervous-system$' OR craniocerebral OR forehead OR 'parietal region' OR 'temporal region' OR 'frontal region' OR occipital) NEAR/3 trauma*):ti,ab,kw | 84858 | 12-May-20 |
| #11 | ((force OR brain OR skull OR blunt OR craniocervical OR 'nervous-system$' OR craniocerebral OR forehead OR 'parietal region' OR 'temporal region' OR 'frontal region' OR occipital) NEAR/3 blast$):ti,ab,kw | 827 | 12-May-20 |
| #10 | ((force OR brain OR skull OR blunt OR craniocervical OR 'nervous-system$' OR craniocerebral OR forehead OR 'parietal region' OR 'temporal region' OR 'frontal region' OR occipital) NEAR/3 lesion$):ti,ab,kw | 32773 | 12-May-20 |
| #9 | ((force OR brain OR skull OR blunt OR craniocervical OR 'nervous-system$' OR craniocerebral OR forehead OR 'parietal region' OR 'temporal region' OR 'frontal region' OR occipital) NEAR/3 defect$):ti,ab,kw | 5535 | 12-May-20 |
| #8 | ((force OR brain OR skull OR blunt OR craniocervical OR 'nervous-system$' OR craniocerebral OR forehead OR 'parietal region' OR 'temporal region' OR 'frontal region' OR occipital) NEAR/3 damag*):ti,ab,kw | 42410 | 12-May-20 |
| #7 | ((force OR brain OR skull OR blunt OR craniocervical OR 'nervous-system$' OR craniocerebral OR forehead OR 'parietal region' OR 'temporal region' OR 'frontal region' OR occipital) NEAR/3 fractur*):ti,ab,kw | 6912 | 12-May-20 |
| #6 | ((force OR brain OR skull OR blunt OR craniocervical OR 'nervous-system$' OR craniocerebral OR forehead OR 'parietal region' OR 'temporal region' OR 'frontal region' OR occipital) NEAR/3 blunt$):ti,ab,kw | 40367 | 12-May-20 |
| #5 | pneumocephal*:ti,ab,kw OR 'pneumo cephal*':ti,ab,kw OR a$rocele*:ti,ab,kw OR pneumocyst*:ti,ab,kw OR 'pneumo cyst*':ti,ab,kw OR (((csf OR cerebrospinal) NEAR/3 (leak* OR otorrhea* OR rhinorrhea*)):ti,ab,kw) OR co$ntrecoup*:ti,ab,kw OR ((co$ntre NEAR/3 coup*):ti,ab,kw) | 28136 | 12-May-20 |
| #4 | pneumocephal*:ti,ab,kw OR 'pneumo cephal*':ti,ab,kw OR a$rocele*:ti,ab,kw OR pneumocyst*:ti,ab,kw OR 'pneumo cyst*':ti,ab,kw OR (((csf OR cerebrospinal) NEAR/3 (leak* OR otorrhea* OR rhinorrhea*)):ti,ab,kw) OR co$ntrecoup*:ti,ab,kw OR ((co$ntre NEAR/3 coup*):ti,ab,kw) | 28136 | 12-May-20 |
| #3 | ibt:ti,ab,kw OR bit:ti,ab,kw OR cti:ti,ab,kw OR bits:ti,ab,kw OR tbi$:ti,ab,kw OR mtbi$:ti,ab,kw OR neurotmes$s:ti,ab,kw OR concussion$:ti,ab,kw OR ((commotio NEXT/1 cerebri):ti,ab,kw) OR contusio*:ti,ab,kw OR ((post NEXT/1 (trauma* OR concussive) NEAR/3 encephalopath*):ti,ab,kw) OR ((brain NEXT/1 laceration$):ti,ab,kw) | 89799 | 12-May-20 |
| #2 | pneumocephal*:ti,ab,kw OR 'pneumo cephal*':ti,ab,kw OR a$rocele*:ti,ab,kw OR pneumocyst*:ti,ab,kw OR 'pneumo cyst*':ti,ab,kw OR (((csf OR cerebrospinal) NEAR/3 (leak* OR otorrhea* OR rhinorrhea*)):ti,ab,kw) OR co$ntrecoup*:ti,ab,kw OR ((co$ntre NEAR/3 coup*):ti,ab,kw) | 28159 | 12-May-20 |
| #1 | pneumocephal*:ti,ab,kw OR 'pneumo cephal*':ti,ab,kw OR a$rocele*:ti,ab,kw OR pneumocyst*:ti,ab,kw OR 'pneumo cyst*':ti,ab,kw OR (((csf OR cerebrospinal) NEAR/3 (leak* OR otorrhea* OR rhinorrhea*)):ti,ab,kw) OR co$ntrecoup*:ti,ab,kw OR ((co$ntre NEAR/3 coup*):ti,ab,kw) | 28159 | 12-May-20 |

***CINAHL- 934***

| **#** | **Query** | **Limiters/Expanders** | **Last Run Via** | **Results** |
| --- | --- | --- | --- | --- |
| S32 | S15 AND S18 AND S31 | Expanders - Apply  equivalent subjects  Search modes -  Boolean/Phrase | Interface - EBSCOhost  Research Databases  Search Screen - Advanced  Search  Database - CINAHL Complete | 934 |
| S31 | S19 OR S20 OR S21 OR  S22 OR S23 OR S24 OR  S25 OR S26 OR S27 OR  S28 OR S29 OR S30 | Expanders - Apply  equivalent subjects  Search modes -  Boolean/Phrase | Interface - EBSCOhost  Research Databases  Search Screen - Advanced  Search  Database - CINAHL Complete | 840,586 |
| S30 | (MH "Intracranial  Hypertension+") | Expanders - Apply  equivalent subjects  Search modes -  Boolean/Phrase | Interface - EBSCOhost  Research Databases  Search Screen - Advanced  Search  Database - CINAHL Complete | 5,482 |
| S29 | (MH "Head Injuries") | Expanders - Apply  equivalent subjects  Search modes -  Boolean/Phrase | Interface - EBSCOhost  Research Databases  Search Screen - Advanced  Search  Database - CINAHL Complete | 7,687 |
| S28 | (MH "Pituitary  Neoplasms+") | Expanders - Apply  equivalent subjects  Search modes -  Boolean/Phrase | Interface - EBSCOhost  Research Databases  Search Screen - Advanced  Search  Database - CINAHL Complete | 1,703 |
| S27 | (MH "Intracranial  Hemorrhage+") | Expanders - Apply  equivalent subjects  Search modes -  Boolean/Phrase | Interface - EBSCOhost  Research Databases  Search Screen - Advanced  Search  Database - CINAHL Complete | 16,158 |
| S26 | (MH "Brain Injuries+") OR  (MH "Skull Fractures+")  OR (MH "Nervous System  Diseases+") | Expanders - Apply  equivalent subjects  Search modes -  Boolean/Phrase | Interface - EBSCOhost  Research Databases  Search Screen - Advanced  Search  Database - CINAHL Complete | 794,917 |
| S25 | (MH "Brain Concussion+") | Expanders - Apply  equivalent subjects  Search modes -  Boolean/Phrase | Interface - EBSCOhost  Research Databases  Search Screen - Advanced  Search  Database - CINAHL Complete | 5,565 |
| S24 | (MH "Craniotomy+") | Expanders - Apply  equivalent subjects  Search modes -  Boolean/Phrase | Interface - EBSCOhost  Research Databases  Search Screen - Advanced  Search  Database - CINAHL Complete | 2,025 |
| S23 | (MH "Neurosurgery+") | Expanders - Apply  equivalent subjects  Search modes -  Boolean/Phrase | Interface - EBSCOhost  Research Databases  Search Screen - Advanced  Search  Database - CINAHL Complete | 30,284 |
| S22 | TI ( (ibt OR bit OR cti OR  bits OR tbi# OR mtbi# OR  neurotmes#s OR  concussion# OR  (commotio W0 cerebri)  OR contusio* OR (post  W0 (trauma* OR  concussive) N2  encephalopath*) OR  (brain W0 laceration#)) )  OR AB ( (ibt OR bit OR cti  OR bits OR tbi# OR mtbi#  OR neurotmes#s OR  concussion# OR  (commotio W0 cerebri)  OR contusio* OR (post  W0 (trauma* OR  concussive) N2  encephalopath*) OR  (brain W0 laceration#)) )  OR MW ( (ibt OR bit OR  cti OR bits OR tbi# OR  mtbi# OR neurotmes#s  OR concussion# OR  (commotio W0 cerebri)  OR contusio* OR (post  W0 (trauma* OR  concussive) N2 encephalopath*) OR  (brain W0 laceration#)) ) | Expanders - Apply  equivalent subjects  Search modes -  Boolean/Phrase | Interface - EBSCOhost  Research Databases  Search Screen - Advanced  Search  Database - CINAHL Complete | 21,953 |
| S21 | TI ( ((force OR brain OR  skull OR blunt OR  craniocervical OR  (nervous W0 system#) OR  craniocerebral OR  forehead OR ((parietal OR  temporal OR frontal) W0  region) OR occipital) N2  (blunt# OR fractur* OR  damag* OR defect# OR  lesion# OR blast# OR  trauma* OR injur* OR  concussion# OR head#)) )  OR AB ( ((force OR brain  OR skull OR blunt OR  craniocervical OR  (nervous W0 system#) OR  craniocerebral OR  forehead OR ((parietal OR  temporal OR frontal) W0  region) OR occipital) N2  (blunt# OR fractur* OR  damag* OR defect# OR  lesion# OR blast# OR  trauma* OR injur* OR  concussion# OR head#)) )  OR MW ( ((force OR brain  OR skull OR blunt OR  craniocervical OR  (nervous W0 system#) OR  craniocerebral OR  forehead OR ((parietal OR  temporal OR frontal) W0  region) OR occipital) N2  (blunt# OR fractur* OR  damag* OR defect# OR  lesion# OR blast# OR  trauma* OR injur* OR  concussion# OR head#)) ) | Expanders - Apply  equivalent subjects  Search modes -  Boolean/Phrase | Interface - EBSCOhost  Research Databases  Search Screen - Advanced  Search  Database - CINAHL Complete | 56,281 |
| S20 | TI ( (pneumocephal* OR  pneumo-cephal* OR  a#rocele* OR  pneumocyst* OR pneumocyst* OR ((CSF  OR cerebrospinal) N2  (leak* OR otorrhea* OR  rhinorrhea*)) OR  co#ntrecoup* OR (co#ntre  N2 coup*)) ) OR AB (  (pneumocephal* OR  pneumo-cephal* OR  a#rocele* OR  pneumocyst* OR  pneumocyst* OR ((CSF  OR cerebrospinal) N2  (leak* OR otorrhea* OR  rhinorrhea*)) OR  co#ntrecoup* OR (co#ntre  N2 coup*)) ) OR MW (  (pneumocephal* OR  pneumo-cephal* OR  a#rocele* OR  pneumocyst* OR  pneumocyst* OR ((CSF  OR cerebrospinal) N2  (leak* OR otorrhea* OR  rhinorrhea*)) OR  co#ntrecoup* OR (co#ntre  N2 coup*)) ) | Expanders - Apply  equivalent subjects  Search modes -  Boolean/Phrase | Interface - EBSCOhost  Research Databases  Search Screen – Advanced  Search  Database - CINAHL Complete | 4,039 |
| S19 | TI ( (neurosurg* OR  neurosurg* OR craniotom*  OR craniectom* OR  trepanation* OR  trepanning* OR  trephination* OR  trephining*) ) OR AB (  (neurosurg* OR  neurosurg* OR craniotom*  OR craniectom* OR  trepanation* OR  trepanning* OR  trephination* OR  trephining*) ) OR MW (  (neurosurg* OR  neurosurg* OR craniotom*  OR craniectom* OR  trepanation* OR  trepanning* OR trephination* OR  trephining*) ) | Expanders - Apply  equivalent subjects  Search modes -  Boolean/Phrase | Interface - EBSCOhost  Research Databases  Search Screen - Advanced  Search  Database - CINAHL Complete | 16,161 |
| S18 | S16 OR S17 | Expanders - Apply  equivalent subjects  Search modes -  Boolean/Phrase | Interface - EBSCOhost  Research Databases  Search Screen - Advanced  Search  Database - CINAHL Complete | 110,310 |
| S17 | MW (middle W2 east*) OR  (north* W2 Africa*) OR  (west* W2 bank) OR  (east* W2 mediterranean)  OR lebanon OR lebanese  OR algeria* OR bahrain*  OR comoros* OR egypt*  OR iraq* OR jordan* OR  kuwait* OR libanaise OR  yemen* OR dubai OR  (abu W2 dhabi) OR aden  OR sanaa OR UAE OR  emirat* OR libya* OR  morocco OR moroccan*  OR ifni OR (trucial W2  state*) OR oman* OR  muscat OR palestin* OR  gaza OR qatar* OR katar*  OR quatar* OR saudi* OR  KSA OR Syria* OR tunis*  OR sudan* OR djibouti*  OR somali* OR  mauritania* OR levant OR  MENA OR EMRO OR  orient OR arabs OR arab  OR arabia) | Expanders - Apply  equivalent subjects  Search modes -  Boolean/Phrase | Interface - EBSCOhost  Research Databases  Search Screen - Advanced  Search  Database - CINAHL Complete | 33,173 |
| S16 | ((MH "Middle East+") OR  (MH "Africa, Northern+"))  OR (TI ((middle W2 east*)  OR (north* W2 Africa*)  OR (west* W2 bank) OR  (east* W2 mediterranean)  OR lebanon OR lebanese  OR algeria* OR bahrain*  OR comoros* OR egypt*  OR iraq* OR jordan* OR  kuwait* OR libanaise OR yemen* OR dubai OR  (abu W2 dhabi) OR aden  OR sanaa OR UAE OR  emirat* OR libya* OR  morocco OR moroccan*  OR ifni OR (trucial W2  state*) OR oman* OR  muscat OR palestin* OR  gaza OR qatar* OR katar*  OR quatar* OR saudi* OR  KSA OR Syria* OR tunis*  OR sudan* OR djibouti*  OR somali* OR  mauritania* OR levant OR  MENA OR EMRO OR  orient OR arabs OR arab  OR arabia) OR AB  ((middle W2 east*) OR  (north* W2 Africa*) OR  (west* W2 bank) OR  (east* W2 mediterranean)  OR lebanon OR lebanese  OR algeria* OR bahrain*  OR comoros* OR egypt*  OR iraq* OR jordan* OR  kuwait* OR libanaise OR  yemen* OR dubai OR  (abu W2 dhabi) OR aden  OR sanaa OR UAE OR  emirat* OR libya* OR  morocco OR moroccan*  OR ifni OR (trucial W2  state*) OR oman* OR  muscat OR palestin* OR  gaza OR qatar* OR katar*  OR quatar* OR saudi* OR  KSA OR Syria* OR tunis*  OR sudan* OR djibouti*  OR somali* OR  mauritania* OR levant OR  MENA OR EMRO OR  orient OR arabs OR arab  OR arabia)) | Expanders - Apply  equivalent subjects  Search modes -  Boolean/Phrase | Interface - EBSCOhost  Research Databases  Search Screen - Advanced  Search  Database - CINAHL Complete | 109,203 |
| S15 | S1 OR S2 OR S3 OR S4  OR S5 OR S6 OR S7 OR  S8 OR S9 OR S10 OR S11 OR S12 OR S13 OR  S14 | Expanders - Apply  equivalent subjects Search modes -  Boolean/Phrase | Interface - EBSCOhost  Research Databases  Search Screen - Advanced Search  Database - CINAHL Complete | 157,092 |
| S14 | (MH "Violence+") | Expanders - Apply  equivalent subjects  Search modes -  Boolean/Phrase | Interface - EBSCOhost  Research Databases  Search Screen - Advanced  Search  Database - CINAHL Complete | 73,424 |
| S13 | (MH "Stress Disorders,  Post-Traumatic+") | Expanders - Apply  equivalent subjects  Search modes -  Boolean/Phrase | Interface - EBSCOhost  Research Databases  Search Screen - Advanced  Search  Database - CINAHL Complete | 24,446 |
| S12 | TI ( ((Adverse W0  Childhood W0  Experience*) OR  ((Domestic OR family OR  gun OR gender-based OR  work* OR expos*) N2  Violence) OR ((child* OR  minor* OR kid# OR  physical* OR elder* OR  spouse* OR partner#) N2  abus*) OR rape OR raping  OR raped OR terroris* OR  bioterroris* OR bioterroris*  OR (mass W0 casualt*  W0 incidence) OR torture  OR (Dowry W0 Death#)) )  OR AB ( ((Adverse W0  Childhood W0  Experience*) OR  ((Domestic OR family OR  gun OR gender-based OR  work* OR expos*) N2  Violence) OR ((child* OR  minor* OR kid# OR  physical* OR elder* OR  spouse* OR partner#) N2  abus*) OR rape OR raping  OR raped OR terroris* OR  bioterroris* OR bioterroris*  OR (mass W0 casualt*  W0 incidence) OR torture  OR (Dowry W0 Death#)) ) OR MW ( ((Adverse W0  Childhood W0  Experience*) OR  ((Domestic OR family OR  gun OR gender-based OR  work* OR expos*) N2  Violence) OR ((child* OR  minor* OR kid# OR  physical* OR elder* OR  spouse* OR partner#) N2  abus*) OR rape OR raping  OR raped OR terroris* OR  bioterroris* OR bioterroris*  OR (mass W0 casualt*  W0 incidence) OR torture  OR (Dowry W0 Death#)) ) | Expanders - Apply  equivalent subjects  Search modes -  Boolean/Phrase | Interface - EBSCOhost  Research Databases  Search Screen - Advanced  Search  Database - CINAHL Complete | 68,581 |
| S11 | TI ( ( assault# OR (Ptss  OR ptsd OR (railway W0  spine) OR ((post-trauma*  OR posttrauma* OR  delayed) W0 stress W0  (disorder# OR syndrome#  OR symptom#))) ) OR AB  ( ( assault# OR (Ptss OR  ptsd OR (railway W0  spine) OR ((post-trauma*  OR posttrauma* OR  delayed) W0 stress W0  (disorder# OR syndrome#  OR symptom#))) ) OR  MW ( ( assault# OR (Ptss  OR ptsd OR (railway W0  spine) OR ((post-trauma*  OR posttrauma* OR  delayed) W0 stress W0  (disorder# OR syndrome#  OR symptom#))) ) | Expanders - Apply  equivalent subjects  Search modes -  Boolean/Phrase | Interface - EBSCOhost  Research Databases  Search Screen - Advanced  Search  Database - CINAHL Complete | 29,032 |
| S10 | TI ( (Stress* N2 (scale#  OR score#)) ) OR AB (  (Stress* N2 (scale# OR  score#)) ) OR MW (  (Stress* N2 (scale# OR  score#)) ) | Expanders - Apply  equivalent subjects  Search modes -  Boolean/Phrase | Interface - EBSCOhost  Research Databases  Search Screen - Advanced  Search  Database - CINAHL Complete | 5,250 |
| S9 | TI ( ((battle* OR war* OR (conflict W0 (zone OR  area)) OR combat OR  combat-related OR  combat-induced OR  combat-associated) AND  (psychiat* OR psycholog*  OR mental* OR stress OR  stressful* OR disorder*  OR fatigue* OR  neuros#s)) ) OR AB (  ((battle* OR war* OR  (conflict W0 (zone OR  area)) OR combat OR  combat-related OR  combat-induced OR  combat-associated) AND  (psychiat* OR psycholog*  OR mental* OR stress OR  stressful* OR disorder*  OR fatigue* OR  neuros#s)) ) OR MW (  ((battle* OR war* OR  (conflict W0 (zone OR  area)) OR combat OR  combat-related OR  combat-induced OR  combat-associated) AND  (psychiat* OR psycholog*  OR mental* OR stress OR  stressful* OR disorder*  OR fatigue* OR  neuros#s)) ) | Expanders - Apply equivalent subjects  Search modes -  Boolean/Phrase | Interface - EBSCOhost Research Databases  Search Screen - Advanced  Search  Database - CINAHL Complete | 28,652 |
| S8 | TI ( ((shell OR mental*)  N1 shock*) ) OR AB (  ((shell OR mental*) N1  shock*) ) OR MW ( ((shell  OR mental*) N1 shock*) ) | Expanders - Apply  equivalent subjects  Search modes -  Boolean/Phrase | Interface - EBSCOhost  Research Databases  Search Screen - Advanced  Search  Database - CINAHL Complete | 57 |
| S7 | TI ( ((war OR hysterical)  W0 neuro*) ) OR AB (  ((war OR hysterical) W0  neuro*) ) OR MW ( ((war  OR hysterical) W0 neuro*)  ) | Expanders - Apply  equivalent subjects  Search modes -  Boolean/Phrase | Interface - EBSCOhost  Research Databases  Search Screen - Advanced  Search  Database - CINAHL Complete | 14 |
| S6 | TI ( ((survivor# OR war* OR hysterical) W0 guilt) )  OR AB ( ((survivor# OR  war* OR hysterical) W0  guilt) ) OR MW (  ((survivor# OR war* OR  hysterical) W0 guilt) ) | Expanders - Apply equivalent subjects  Search modes -  Boolean/Phrase | Interface - EBSCOhost Research Databases  Search Screen - Advanced  Search  Database - CINAHL Complete | 41 |
| S5 | TI ( (recurren* N2  (thought# OR image#)) )  OR AB ( (recurren* N2  (thought# OR image#)) )  OR MW ( (recurren* N2  (thought# OR image#)) ) | Expanders - Apply  equivalent subjects  Search modes -  Boolean/Phrase | Interface - EBSCOhost  Research Databases  Search Screen - Advanced  Search  Database - CINAHL Complete | 127 |
| S4 | TI ( (psycholog* N2 (scar#  OR suffering)) ) OR AB (  (psycholog* N2 (scar# OR  suffering)) ) OR MW (  (psycholog* N2 (scar# OR  suffering)) ) | Expanders - Apply  equivalent subjects  Search modes -  Boolean/Phrase | Interface - EBSCOhost  Research Databases  Search Screen - Advanced  Search  Database - CINAHL Complete | 369 |
| S3 | TI ( (psycholog* N3  ((witness* OR experienc*)  W0 shock*)) ) OR AB (  (psycholog* N3 ((witness*  OR experienc*) W0  shock*)) ) OR MW (  (psycholog* N3 ((witness*  OR experienc*) W0  shock*)) ) | Expanders - Apply  equivalent subjects  Search modes -  Boolean/Phrase | Interface - EBSCOhost  Research Databases  Search Screen - Advanced  Search  Database - CINAHL Complete | 1 |
| S2 | TI ( (illness AND (frighten*  OR fight* OR war OR  combat* OR battle*)) ) OR  AB ( (illness AND  (frighten* OR fight* OR  war OR combat* OR  battle*)) ) OR MW (  (illness AND (frighten* OR  fight* OR war OR combat*  OR battle*)) ) | Expanders - Apply  equivalent subjects  Search modes -  Boolean/Phrase | Interface - EBSCOhost  Research Databases  Search Screen - Advanced  Search  Database - CINAHL Complete | 1,475 |
| S1 | TI ( ((((child* OR female#  OR male# OR m#n OR  wom#n OR adult# OR  boy# OR girl# OR  adolescent# OR old OR  elderly OR past) W0 trauma#) OR traumatic  OR War* OR Battle* OR  Combat# OR Post-  Traumatic OR  posttraumatic OR  combatrelated OR  combatassociated OR  combatinduced OR postcombat)  AND (mental* OR  psychiat* OR psycholog*  OR psychic OR ideation#  OR idea# OR thought#  OR stress OR stressful  OR stresses)) ) OR AB (  ((((child* OR female# OR  male# OR m#n OR  wom#n OR adult# OR  boy# OR girl# OR  adolescent# OR old OR  elderly OR past) W0  trauma#) OR traumatic  OR War* OR Battle* OR  Combat# OR Post-  Traumatic OR  posttraumatic OR  combatrelated OR  combatassociated OR  combatinduced OR postcombat)  AND (mental* OR  psychiat* OR psycholog*  OR psychic OR ideation#  OR idea# OR thought#  OR stress OR stressful  OR stresses)) ) OR MW (  ((((child* OR female# OR  male# OR m#n OR  wom#n OR adult# OR  boy# OR girl# OR  adolescent# OR old OR  elderly OR past) W0  trauma#) OR traumatic  OR War* OR Battle* OR  Combat# OR Post-  Traumatic OR  posttraumatic OR  combatrelated OR combatassociated OR  combatinduced OR postcombat)  AND (mental* OR  psychiat* OR psycholog*  OR psychic OR ideation#  OR idea# OR thought#  OR stress OR stressful  OR stresses)) ) | Expanders - Apply  equivalent subjects  Search modes -  Boolean/Phrase | Interface - EBSCOhost  Research Databases  Search Screen - Advanced  Search  Database - CINAHL Complete | 55,457 |

***PsycInfo- 1,644***

| **#** | **Query** | **Limiters/Expanders** | **Last Run Via** | **Results** |
| --- | --- | --- | --- | --- |
| S24 | S15 AND S16 AND S23 | Expanders - Apply  equivalent subjects  Search modes -  Boolean/Phrase | Interface - EBSCOhost  Research Databases  Search Screen - Basic Search  Database – APA PsycInfo | 1,644 |
| S23 | S17 OR S18 OR S19 OR  S20 OR S21 OR S22 | Expanders - Apply  equivalent subjects  Search modes -  Boolean/Phrase | Interface - EBSCOhost  Research Databases  Search Screen - Basic Search  Database - APA PsycInfo | 205,550 |
| S22 | (((((DE "Neurosurgery")  OR (DE "Trauma" OR DE  "Birth Trauma" OR DE  "Emotional Trauma" OR  DE "Injuries" OR DE  "Moral Injury" OR DE  "Post-Traumatic Stress"  OR DE "Posttraumatic  Growth" OR DE  "Traumatic Brain Injury"  OR DE "Traumatic Loss"  OR DE "Head Injuries"  OR DE "Brain  Concussion" OR DE  "Nervous System  Disorders" OR DE  "Autonomic Nervous  System Disorders" OR DE  "Central Nervous System  Disorders" OR DE  "Charcot-Marie-Tooth  Disease" OR DE  "Demyelination" OR DE  "Dyspraxia" OR DE "Head  Injuries" OR DE  "Hyperkinesis" OR DE  "Locked-In Syndrome" OR  DE "Meningoradiculitis"  OR DE "Movement  Disorders" OR DE "Nervous System  Neoplasms" OR DE  "Neurodegenerative  Diseases" OR DE  "Neurofibromatosis" OR  DE "Neuroinflammation"  OR DE "Neuromuscular  Disorders" OR DE  "Neuropathy" OR DE  "Perceptual Disturbances"  OR DE "Restless Leg  Syndrome" OR DE  "Sclerosis (Nervous  System)" OR DE  "Seizures" OR DE "Sleep  Wake Disorders" OR DE  "Brain Injuries" OR DE  "Traumatic Brain Injury"  OR DE "Traumatic Brain  Injury" OR DE "Brain  Concussion")) OR (DE  "Hematoma")) OR (DE  "Subarachnoid  Hemorrhage")) OR (DE  "Pituitary Disorders" OR  DE "Hypopituitarism")) OR  (DE "Intracranial  Abscesses") | Expanders - Apply  equivalent subjects  Search modes -  Boolean/Phrase | Interface - EBSCOhost  Research Databases  Search Screen - Basic Search  Database - APA PsycInfo | 151,431 |
| S21 | TI ( (ibt OR bit OR cti OR  bits OR tbi# OR mtbi# OR  neurotmes#s OR  concussion# OR  (commotio W0 cerebri)  OR contusio* OR (post  W0 (trauma* OR  concussive) N2  encephalopath*) OR  (brain W0 laceration#)) )  OR AB ( (ibt OR bit OR cti  OR bits OR tbi# OR mtbi#  OR neurotmes#s OR  concussion# OR  (commotio W0 cerebri)  OR contusio* OR (post  W0 (trauma* OR  concussive) N2 encephalopath*) OR  (brain W0 laceration#)) )  OR SU ( (ibt OR bit OR cti  OR bits OR tbi# OR mtbi#  OR neurotmes#s OR  concussion# OR  (commotio W0 cerebri)  OR contusio* OR (post  W0 (trauma* OR  concussive) N2  encephalopath*) OR  (brain W0 laceration#)) ) | Expanders - Apply  equivalent subjects  Search modes -  Boolean/Phrase | Interface - EBSCOhost  Research Databases  Search Screen - Basic Search  Database - APA PsycInfo | 21,087 |
| S20 | TI ( ((force OR brain OR  skull OR blunt OR  craniocervical OR  (nervous W0 system#) OR  craniocerebral OR  forehead OR ((parietal OR  temporal OR frontal) W0  region) OR occipital) N2  (blunt# OR fractur* OR  damag* OR defect# OR  lesion# OR blast# OR  trauma* OR injur* OR  concussion# OR head#)) )  OR AB ( ((force OR brain  OR skull OR blunt OR  craniocervical OR  (nervous W0 system#) OR  craniocerebral OR  forehead OR ((parietal OR  temporal OR frontal) W0  region) OR occipital) N2  (blunt# OR fractur* OR  damag* OR defect# OR  lesion# OR blast# OR  trauma* OR injur* OR  concussion# OR head#)) )  OR SU ( ((force OR brain  OR skull OR blunt OR  craniocervical OR  (nervous W0 system#) OR  craniocerebral OR  forehead OR ((parietal OR  temporal OR frontal) W0  region) OR occipital) N2 (blunt# OR fractur* OR  damag* OR defect# OR  lesion# OR blast# OR  trauma* OR injur* OR  concussion# OR head#)) ) | Expanders - Apply  equivalent subjects  Search modes -  Boolean/Phrase | Interface - EBSCOhost  Research Databases  Search Screen - Basic Search  Database - APA PsycInfo | 65,659 |
| S19 | TI ( (pneumocephal* OR  pneumo-cephal* OR  a#rocele* OR  pneumocyst* OR pneumocyst*  OR ((CSF OR  cerebrospinal) N2 (leak*  OR otorrhea* OR  rhinorrhea*)) OR  co#ntrecoup* OR (co#ntre  N2 coup*)) ) OR AB (  (pneumocephal* OR  pneumo-cephal* OR  a#rocele* OR  pneumocyst* OR pneumocyst*  OR ((CSF OR  cerebrospinal) N2 (leak*  OR otorrhea* OR  rhinorrhea*)) OR  co#ntrecoup* OR (co#ntre  N2 coup*)) ) OR SU (  (pneumocephal* OR  pneumo-cephal* OR  a#rocele* OR  pneumocyst* OR pneumocyst*  OR ((CSF OR  cerebrospinal) N2 (leak*  OR otorrhea* OR  rhinorrhea*)) OR  co#ntrecoup* OR (co#ntre  N2 coup*)) ) | Expanders - Apply  equivalent subjects  Search modes -  Boolean/Phrase | Interface - EBSCOhost  Research Databases  Search Screen - Basic Search  Database - APA PsycInfo | 296 |
| S18 | TI ( (neurosurg* OR  neuro-surg* OR  craniotom* OR  craniectom* OR  trepanation* OR  trepanning* OR  trephination* OR  trephining*) ) OR AB (  (neurosurg* OR neurosurg*  OR craniotom* OR craniectom* OR  trepanation* OR  trepanning* OR  trephination* OR  trephining*) ) OR SU (  (neurosurg* OR neurosurg*  OR craniotom* OR  craniectom* OR  trepanation* OR  trepanning* OR  trephination* OR  trephining*) ) | Expanders - Apply  equivalent subjects  Search modes -  Boolean/Phrase | Interface - EBSCOhost  Research Databases  Search Screen - Basic Search  Database - APA PsycInfo | 6,864 |
| S17 | TI ( ((brain* OR  encephalon* OR cranium  OR cranio* OR cranial OR  intracrani* OR intra-crani*  OR skull* OR cerebral OR  cerebell* OR head OR  ventric* OR hemispher*  OR pontine OR putamin*  OR dura* OR subdura*  OR sub-dura* OR ((supra  OR extra) N1 dura*) OR  supradura* OR epidura*  OR epi-dura* OR  arachnoid* OR subarachnoid*  OR  subarachnoid* OR (intra  N1 arachnoid*)) N2 (injur*  OR trauma* OR  concussion* OR postconcussion*  OR  h#emorrhag* OR  h#ematom* OR bleed* OR  penetrat* OR (non N1  penetrat*) OR edema* OR  oedema* OR fracture* OR  aneurysm* OR pressur*  OR lesion* OR swell* OR  contusion*)) ) OR AB (  ((brain* OR encephalon*  OR cranium OR cranio*  OR cranial OR intracrani*  OR intra-crani* OR skull*  OR cerebral OR cerebell*  OR head OR ventric* OR hemispher* OR pontine  OR putamin* OR dura*  OR subdura* OR subdura*  OR ((supra OR  extra) N1 dura*) OR  supradura* OR epidura*  OR epi-dura* OR  arachnoid* OR subarachnoid*  OR  subarachnoid* OR (intra  N1 arachnoid*)) N2 (injur*  OR trauma* OR  concussion* OR postconcussion*  OR  h#emorrhag* OR  h#ematom* OR bleed* OR  penetrat* OR (non N1  penetrat*) OR edema* OR  oedema* OR fracture* OR  aneurysm* OR pressur*  OR lesion* OR swell* OR  contusion*)) ) OR SU (  ((brain* OR encephalon*  OR cranium OR cranio*  OR cranial OR intracrani*  OR intra-crani* OR skull*  OR cerebral OR cerebell*  OR head OR ventric* OR  hemispher* OR pontine  OR putamin* OR dura*  OR subdura* OR subdura*  OR ((supra OR  extra) N1 dura*) OR  supradura* OR epidura*  OR epi-dura* OR  arachnoid* OR subarachnoid*  OR  subarachnoid* OR (intra  N1 arachnoid*)) N2 (injur*  OR trauma* OR  concussion* OR postconcussion*  OR  h#emorrhag* OR  h#ematom* OR bleed* OR  penetrat* OR (non N1  penetrat*) OR edema* OR oedema* OR fracture* OR  aneurysm* OR pressur*  OR lesion* OR swell* OR  contusion*)) ) | Expanders - Apply  equivalent subjects  Search modes -  Boolean/Phrase | Interface - EBSCOhost  Research Databases  Search Screen - Basic Search  Database - APA PsycInfo | 60,542 |
| S16 | TI ( (middle W2 east*) OR  (north* W2 Africa*) OR  (east* W2 mediterranean)  OR lebanon OR lebanese  OR algeria* OR bahrain*  OR egypt* OR iraq* OR  jordan* OR kuwait* OR  libanaise OR yemen* OR  dubai OR (abu W2 dhabi)  OR aden OR sanaa OR  UAE OR emirat* OR libya*  OR morocco OR  moroccan* OR ifni OR  (trucial W2 state) OR  (west* W2 bank) OR  oman* OR muscat OR  palestin* OR gaza OR  qatar* OR katar* OR  quatar* OR saudi* OR  KSA OR Syria* OR  comoros* OR tunis* OR  levant OR MENA OR  EMRO OR orient OR  arabs OR arab OR arabia  ) OR AB ( (middle W2  east*) OR (north* W2  Africa*) OR (east* W2  mediterranean) OR  lebanon OR lebanese OR  algeria* OR bahrain* OR  egypt* OR iraq* OR  jordan* OR kuwait* OR  libanaise OR yemen* OR  dubai OR (abu W2 dhabi)  OR aden OR sanaa OR  UAE OR emirat* OR libya*  OR morocco OR  moroccan* OR ifni OR  (trucial W2 state) OR  (west* W2 bank) OR  oman* OR muscat OR  palestin* OR gaza OR qatar* OR katar* OR  quatar* OR saudi* OR  KSA OR Syria* OR  comoros* OR tunis* OR  levant OR MENA OR  EMRO OR orient OR  arabs OR arab OR arabia  ) OR SU ( (middle W2  east*) OR (north* W2  Africa*) OR (east* W2  mediterranean) OR  lebanon OR lebanese OR  algeria* OR bahrain* OR  egypt* OR iraq* OR  jordan* OR kuwait* OR  libanaise OR yemen* OR  dubai OR (abu W2 dhabi)  OR aden OR sanaa OR  UAE OR emirat* OR libya*  OR morocco OR  moroccan* OR ifni OR  (trucial W2 state) OR  (west* W2 bank) OR  oman* OR muscat OR  palestin* OR gaza OR  qatar* OR katar* OR  quatar* OR saudi* OR  KSA OR Syria* OR  comoros* OR tunis* OR  levant OR MENA OR  EMRO OR orient OR  arabs OR arab OR arabia  ) | Expanders - Apply  equivalent subjects  Search modes -  Boolean/Phrase | Interface - EBSCOhost  Research Databases  Search Screen - Basic Search  Database - APA PsycInfo | 34,770 |
| S15 | S1 OR S2 OR S3 OR S4  OR S5 OR S6 OR S7 OR  S8 OR S9 OR S10 OR  S11 OR S12 OR S13 OR  S14 | Expanders - Apply  equivalent subjects  Search modes -  Boolean/Phrase | Interface - EBSCOhost  Research Databases  Search Screen - Basic Search  Database - APA PsycInfo | 246,530 |
| S14 | DE "Violence" OR DE  "Dating Violence" OR DE  "Domestic Violence" OR  DE "Gun Violence" OR  DE "Intimate Partner  Violence" OR DE "Patient  Violence" OR DE "Political Violence" OR DE "School  Violence" OR DE "Violent  Crime" OR DE  "Workplace Violence" | Expanders - Apply  equivalent subjects  Search modes -  Boolean/Phrase | Interface - EBSCOhost  Research Databases  Search Screen - Basic Search  Database - APA PsycInfo | 60,534 |
| S13 | DE "Posttraumatic Stress  Disorder" OR DE  "Complex PTSD" OR DE  "DESNOS" | Expanders - Apply  equivalent subjects  Search modes -  Boolean/Phrase | Interface - EBSCOhost  Research Databases  Search Screen - Basic Search  Database - APA PsycInfo | 32,607 |
| S12 | TI ( ((Adverse W0  Childhood W0  Experience*) OR  ((Domestic OR family OR  gun OR gender-based OR  work* OR expos*) N2  Violence) OR ((child* OR  minor* OR kid# OR  physical* OR elder* OR  spouse* OR partner#) N2  abus*) OR rape OR raping  OR raped OR terroris* OR  bioterroris* OR bioterroris*  OR (mass W0  casualt* W0 incidence)  OR torture OR (Dowry W0  Death#)) ) OR AB (  ((Adverse W0 Childhood  W0 Experience*) OR  ((Domestic OR family OR  gun OR gender-based OR  work* OR expos*) N2  Violence) OR ((child* OR  minor* OR kid# OR  physical* OR elder* OR  spouse* OR partner#) N2  abus*) OR rape OR raping  OR raped OR terroris* OR  bioterroris* OR bioterroris*  OR (mass W0  casualt* W0 incidence)  OR torture OR (Dowry W0  Death#)) ) OR SU (  ((Adverse W0 Childhood  W0 Experience*) OR  ((Domestic OR family OR gun OR gender-based OR  work* OR expos*) N2  Violence) OR ((child* OR  minor* OR kid# OR  physical* OR elder* OR  spouse* OR partner#) N2  abus*) OR rape OR raping  OR raped OR terroris* OR  bioterroris* OR bioterroris*  OR (mass W0  casualt* W0 incidence)  OR torture OR (Dowry W0  Death#)) ) | Expanders - Apply  equivalent subjects  Search modes -  Boolean/Phrase | Interface - EBSCOhost  Research Databases  Search Screen - Basic Search  Database - APA PsycInfo | 95,683 |
| S11 | TI ( assault# OR (Ptss OR  ptsd OR (railway W0  spine) OR ((post-trauma*  OR posttrauma* OR  delayed) W0 stress W0  (disorder# OR syndrome#  OR symptom#))) ) OR AB  ( assault# OR (Ptss OR  ptsd OR (railway W0  spine) OR ((post-trauma*  OR posttrauma* OR  delayed) W0 stress W0  (disorder# OR syndrome#  OR symptom#))) ) OR SU  ( assault# OR (Ptss OR  ptsd OR (railway W0  spine) OR ((post-trauma*  OR posttrauma* OR  delayed) W0 stress W0  (disorder# OR syndrome#  OR symptom#))) ) | Expanders - Apply  equivalent subjects  Search modes -  Boolean/Phrase | Interface - EBSCOhost  Research Databases  Search Screen - Basic Search  Database - APA PsycInfo | 56,695 |
| S10 | TI ( (Stress* N2 (scale#  OR score#)) ) OR AB (  (Stress* N2 (scale# OR  score#)) ) OR SU (  (Stress* N2 (scale# OR  score#)) ) | Expanders - Apply  equivalent subjects  Search modes -  Boolean/Phrase | Interface - EBSCOhost  Research Databases  Search Screen - Basic Search  Database - APA PsycInfo | 7,198 |
| S9 | TI ( ((battle* OR war* OR  (conflict W0 (zone OR  area)) OR combat OR  combat-related OR combat-induced OR  combat-associated) AND  (psychiat* OR psycholog*  OR mental* OR stress OR  stressful* OR disorder*  OR fatigue* OR  neuros#s)) ) OR AB (  ((battle* OR war* OR  (conflict W0 (zone OR  area)) OR combat OR  combat-related OR  combat-induced OR  combat-associated) AND  (psychiat* OR psycholog*  OR mental* OR stress OR  stressful* OR disorder*  OR fatigue* OR  neuros#s)) ) OR SU (  ((battle* OR war* OR  (conflict W0 (zone OR  area)) OR combat OR  combat-related OR  combat-induced OR  combat-associated) AND  (psychiat* OR psycholog*  OR mental* OR stress OR  stressful* OR disorder*  OR fatigue* OR  neuros#s)) ) | Expanders - Apply  equivalent subjects  Search modes -  Boolean/Phrase | Interface - EBSCOhost  Research Databases  Search Screen - Basic Search  Database - APA PsycInfo | 59,643 |
| S8 | TI ( ((shell OR mental*)  N1 shock*) ) OR AB (  ((shell OR mental*) N1  shock*) ) OR SU ( ((shell  OR mental*) N1 shock*) ) | Expanders - Apply  equivalent subjects  Search modes -  Boolean/Phrase | Interface - EBSCOhost  Research Databases  Search Screen - Basic Search  Database - APA PsycInfo | 252 |
| S7 | TI ( ((war OR hysterical)  W0 neuro*) ) OR AB (  ((war OR hysterical) W0  neuro*) ) OR SU ( ((war  OR hysterical) W0 neuro*)  ) | Expanders - Apply  equivalent subjects  Search modes -  Boolean/Phrase | Interface - EBSCOhost  Research Databases  Search Screen - Basic Search  Database - APA PsycInfo | 577 |
| S6 | TI ( ((survivor# OR war*  OR hysterical) W0 guilt) )  OR AB ( ((survivor# OR  war* OR hysterical) W0 guilt) ) OR SU (  ((survivor# OR war* OR  hysterical) W0 guilt) ) | Expanders - Apply  equivalent subjects  Search modes -  Boolean/Phrase | Interface - EBSCOhost  Research Databases  Search Screen - Basic Search  Database - APA PsycInfo | 222 |
| S5 | TI ( (recurren* N2  (thought# OR image#)) )  OR AB ( (recurren* N2  (thought# OR image#)) )  OR SU ( (recurren* N2  (thought# OR image#)) ) | Expanders - Apply  equivalent subjects  Search modes -  Boolean/Phrase | Interface - EBSCOhost  Research Databases  Search Screen - Basic Search  Database - APA PsycInfo | 274 |
| S4 | TI ( (psycholog* N2 (scar#  OR suffering)) ) OR AB (  (psycholog* N2 (scar# OR  suffering)) ) OR SU (  (psycholog* N2 (scar# OR  suffering)) ) | Expanders - Apply  equivalent subjects  Search modes -  Boolean/Phrase | Interface - EBSCOhost  Research Databases  Search Screen - Basic Search  Database - APA PsycInfo | 891 |
| S3 | TI ( (psycholog* N3  ((witness* OR experienc*)  W0 shock*)) ) OR AB (  (psycholog* N3 ((witness*  OR experienc*) W0  shock*)) ) OR SU (  (psycholog* N3 ((witness*  OR experienc*) W0  shock*)) ) | Expanders - Apply  equivalent subjects  Search modes -  Boolean/Phrase | Interface - EBSCOhost  Research Databases  Search Screen - Basic Search  Database - APA PsycInfo | 1 |
| S2 | TI ( (illness AND (frighten*  OR fight* OR war OR  combat* OR battle*)) ) OR  AB ( (illness AND  (frighten* OR fight* OR  war OR combat* OR  battle*)) ) OR SU ( (illness  AND (frighten* OR fight*  OR war OR combat* OR  battle*)) ) | Expanders - Apply  equivalent subjects  Search modes -  Boolean/Phrase | Interface - EBSCOhost  Research Databases  Search Screen - Basic Search  Database - APA PsycInfo | 2,805 |
| S1 | TI ( ((((child* OR female#  OR male# OR m#n OR  wom#n OR adult# OR  boy# OR girl# OR  adolescent# OR old OR  elderly OR past) W0  trauma#) OR traumatic  OR War* OR Battle* OR  Combat# OR Post- Traumatic OR  posttraumatic OR combatrelated  OR combatassociated  OR combatinduced  OR post-combat)  AND (mental* OR  psychiat* OR psycholog*  OR psychic OR ideation#  OR idea# OR thought#  OR stress OR stressful  OR stresses)) ) OR AB (  ((((child* OR female# OR  male# OR m#n OR  wom#n OR adult# OR  boy# OR girl# OR  adolescent# OR old OR  elderly OR past) W0  trauma#) OR traumatic  OR War* OR Battle* OR  Combat# OR Post-  Traumatic OR  posttraumatic OR combatrelated  OR combatassociated  OR combatinduced  OR post-combat)  AND (mental* OR  psychiat* OR psycholog*  OR psychic OR ideation#  OR idea# OR thought#  OR stress OR stressful  OR stresses))) OR SU (  ((((child* OR female# OR  male# OR m#n OR  wom#n OR adult# OR  boy# OR girl# OR  adolescent# OR old OR  elderly OR past) W0  trauma#) OR traumatic  OR War* OR Battle* OR  Combat# OR Post-  Traumatic OR  posttraumatic OR combatrelated  OR combatassociated  OR combatinduced  OR post-combat)  AND (mental* OR psychiat* OR psycholog*  OR psychic OR ideation#  OR idea# OR thought#  OR stress OR stressful  OR stresses)) ) | Expanders - Apply  equivalent subjects  Search modes -  Boolean/Phrase | Interface - EBSCOhost  Research Databases  Search Screen - Basic Search  Database - APA PsycInfo | 114,971 |
